# Distribution of dipeptide repeat proteins in cellular models and *C9orf72* mutation cases suggests link to transcriptional silencing

**DOI:** 10.1007/s00401-015-1450-z

**Published:** 2015-06-18

**Authors:** Martin H. Schludi, Stephanie May, Friedrich A. Grässer, Kristin Rentzsch, Elisabeth Kremmer, Clemens Küpper, Thomas Klopstock, German Consortium for Frontotemporal Lobar Degeneration, Bavarian Brain Banking Alliance, Thomas Arzberger, Dieter Edbauer

**Affiliations:** German Center for Neurodegenerative Diseases (DZNE), Feodor-Lynen-Str. 17, 81337 Munich, Germany; Munich Cluster of Systems Neurology (SyNergy), Munich, Germany; Institute of Virology, Saarland University Medical School, 66421 Homburg, Germany; Institute of Molecular Immunology, Helmholtz Zentrum München, German Research Center for Environmental Health (GmbH), Marchioninistr. 25, 81377 Munich, Germany; Department of Neurology, Friedrich-Baur-Institute, Ludwig-Maximilians-University, 80336 Munich, Germany; Center for Neuropathology and Prion Research, Ludwig-Maximilians-University Munich, Feodor-Lynen-Str. 23, 81377 Munich, Germany; Department of Psychiatry and Psychotherapy, Ludwig-Maximilians University Munich, Nußbaumstraße 7, 80336 Munich, Germany; Institute for Metabolic Biochemistry, Ludwig-Maximilians University Munich, 81377 Munich, Germany

**Keywords:** ALS, FTLD, Repeat disorders, C9orf72, DPR inclusions, Neurotoxicity

## Abstract

A massive expansion of a GGGGCC repeat upstream of the *C9orf72* coding region is the most common known cause of amyotrophic lateral sclerosis and frontotemporal dementia. Despite its intronic localization and lack of a canonical start codon, both strands are translated into aggregating dipeptide repeat (DPR) proteins: poly-GA, poly-GP, poly-GR, poly-PR and poly-PA. To address conflicting findings on the predominant toxicity of the different DPR species in model systems, we compared the expression pattern of the DPR proteins in rat primary neurons and postmortem brain and spinal cord of *C9orf72* mutation patients. Only poly-GA overexpression closely mimicked the p62-positive neuronal cytoplasmic inclusions commonly observed for all DPR proteins in patients. In contrast, overexpressed poly-GR and poly-PR formed nucleolar p62-negative inclusions. In patients, most of the less common neuronal intranuclear DPR inclusions were para-nucleolar and p62 positive. Neuronal nucleoli in *C9orf72* cases showed normal size and morphology regardless of the presence of poly-GR and poly-PR inclusions arguing against widespread nucleolar stress, reported in cellular models. Colocalization of para-nucleolar DPR inclusions with heterochromatin and a marker of transcriptional repression (H3K9me2) indicates a link to gene transcription. In contrast, we detected numerous intranuclear DPR inclusions not associated with nucleolar structures in ependymal and subependymal cells. In patients, neuronal inclusions of poly-GR, poly-GP and the poly-GA interacting protein Unc119 were less abundant than poly-GA inclusions, but showed similar regional and subcellular distribution. Regardless of neurodegeneration, all inclusions were most abundant in neocortex, hippocampus and thalamus, with few inclusions in brain stem and spinal cord. In the granular cell layer of the cerebellum, poly-GA and Unc119 inclusions were significantly more abundant in cases with FTLD than in cases with MND and FTLD/MND. Poly-PR inclusions were rare throughout the brain but significantly more abundant in the CA3/4 region of FTLD cases than in MND cases. Thus, although DPR distribution is not correlated with neurodegeneration spatially, it correlates with neuropathological subtypes.

**Electronic supplementary material:**

The online version of this article (doi:10.1007/s00401-015-1450-z) contains supplementary material, which is available to authorized users.

## Introduction

About 10 % of all patients with amyotrophic lateral sclerosis (ALS), frontotemporal dementia (FTD) or mixed presentation of both diseases (ALS/FTD) are caused by a massive expansion of a GGGGCC repeat upstream of the *C9orf72*-coding region [11, 18, 43]. Three main hypotheses have been proposed to explain the pathomechanism of *C9orf72* disease. First, reduced expression of the mutant allele suggests a loss of function mechanism [11, 18]. Studies in *C. elegans* and zebrafish reported motor deficits [7, 51], although loss of *C9orf72* has no obvious effect in cultured neurons and mice [25, 55]. Second, the repeat RNA may induce toxicity by sequestering endogenous RNA-binding proteins in nuclear RNA foci [16]. A large number of GGGGCC-interacting proteins have been identified, but their contribution to *C9orf72* disease has not been elucidated so far [9, 27, 37]. Additionally, formation of RNA·DNA hybrids of the expanded repeat (so-called R-loops) may contribute to toxicity by interfering with transcription [20, 54]. However, in cultured primary neurons and the fly retina even high-level expression of repeat RNA causes little or no toxicity [35, 55]. Third, although located in an intron and lacking an ATG start codon, sense and antisense transcripts of the expanded repeat are translated by an unconventional mechanism into five dipeptide repeat (DPR) protein species [1, 17, 36, 38, 60]. All DPR species are detected in neuronal inclusions throughout the central nervous system (CNS) of *C9orf72* mutation patients, predominantly in the cytoplasm. Inclusions of poly-(glycine–alanine) (poly-GA), poly-(glycine–arginine) (poly-GR) and poly-(glycine–proline) (poly-GP) proteins encoded by the sense strand are far more abundant than poly-(proline–alanine) (poly-PA) and poly-(proline–arginine) (poly-PR) proteins encoded by the antisense strand [17, 36]. None of these mechanisms, however, has so far explained the origin of neuronal and glial TDP-43 inclusions found in almost all cases with *C9orf72* mutation, and the variable expression of dementia and motor symptoms even within the same family [16, 33]. Interestingly, the first clinical symptoms and neurodegeneration seem to arise prior to the onset of TDP-43 pathology when DPR inclusion pathology is already widespread [2, 36, 38, 42].

Recently, several groups reported toxicity of recombinantly expressed individual DPR species in cell lines, primary neurons and the fly retina. This led to a controversy about the main toxic DPR species. Several groups showed neurotoxicity of poly-GA, the most abundant DPR inclusion protein in *C9orf72* mutation patients. Poly-GA toxicity has been attributed to co-aggregation of the transport factor Unc119 [34] and impairment of the proteasome [57, 59]. However, in contrast to TDP-43 inclusions, poly-GA inclusions show no spatial correlation with neurodegeneration in patients [10, 29]. Other reports favor toxicity of the arginine-rich DPR species, poly-GR and poly-PR, by interference with global RNA metabolism and protein synthesis [23, 35, 55]. While poly-GR and poly-PR localization was not analyzed in the fly model [35], cell culture studies found overexpressed poly-GR and poly-PR (20–400 repeats) predominantly in nucleolar aggregates [23, 34, 55, 57, 59]. This is in strong contrast to the predominantly cytoplasmic localization of poly-GR and poly-PR described in patients so far [17, 36, 38, 60]. Poly-GP also has been reported to induce toxicity in cell lines, although no mechanism was proposed [60]. Only poly-PA was not toxic in any system tested. However, none of the proposed pathomechanisms has been rigorously validated in patient tissue.

Prompted by conflicting reports on the neurotoxicity of DPR proteins in vitro, we carefully compared the expression of recombinant DPR proteins in primary rat neurons of all DPR species with proposed neurotoxicity, including the predominant sense strand-derived DPR inclusions and poly-PR, in patient brain using novel monoclonal antibodies particularly focusing on nuclear and nucleolar pathology. Since toxic overexpressed arginine-rich DPRs mainly aggregate in p62-negative intranuclear inclusions, we tried to identify such inclusions in key areas of neurodegeneration in patient CNS. Additionally, we analyzed the regional distribution pattern of aggregates containing poly-GA, its interacting partner Unc119, poly-GR, poly-GP or poly-PR in brain and spinal cord of autopsy cases with *C9orf72* mutation and correlated aggregate frequency with the neuropathological diagnosis.

## Materials and methods

### Antibodies and reagents

The following antibodies were used: anti-nucleolin (rabbit polyclonal and mouse monoclonal, Abcam, Cambridge England), anti-p62/SQSTM1 (rabbit polyclonal, MBL, Nagoya Japan and mouse monoclonal, BD, Belgium), anti-poly-GA clone 5E9 (mouse monoclonal) [29], anti-Unc119 (rabbit polyclonal, homemade) [34], anti-fibrillarin (rabbit polyclonal, Abcam), anti-GST (rabbit polyclonal, Eurogentec, Belgium), anti-H3K9me2 (Cell Signaling Technology, Cambridge, England), anti-HDAC6 (Santa Cruz, Dallas, Texas), anti-CUG-BP1 (Abcam), anti-PML (Abcam), anti-HSF1 (Santa Cruz), anti-CD99/MIC2 (Thermo scientific, Waltham, Massachusetts), anti-PSMC2 and anti-PSMC4 (Bethyl laboratories, Montgomery, Texas), anti-Coilin (Abcam) and anti-p53 (Ventana, Tuscon, Arizona). Poly-GR antibodies 5A2 and 5H9 have been described previously [36, 38]. The novel poly-GR-specific clone 7H1 (rat isotype IgG2c) was identified by rescreening monoclonal antibodies raised against the EBNA2 epitope GQSRGRGRGRGRGRGKGKSRDK with asymmetrically dimethylated arginines [19] and screened by ELISA against biotinylated (GR)_10_ peptides (Peps4LifeSciences, Heidelberg, Germany) as described [36]. Like clone 5H9, 7H1 detected (GR)_10_ with asymmetrically dimethylated arginines and non-methylated arginines, but also weakly cross-reacts with (GR)_10_ containing symmetrically dimethylated arginines (data not shown). By immunizing rats with synthetic GP_10_ peptides the poly-GP-specific antibody 7A5 (isotype IgG2c) was raised using previously described protocols [29]. Poly-PR antibody 32B3 (isotype IgG2b) was raised against synthetic PR_10_ peptides in mouse using the same protocol.

RNA was stained with SYTO12 and SYTO RNAselect (Life Technologies, Darmstadt, Germany) and nuclei were stained with DAPI (Roche Applied Science, Penzberg, Germany).

### DNA constructs and lentivirus production

Previously described cDNAs of GA_175_-GFP and GFP-GR_149_, GP_80_-V5/His and PR_175_-GFP with ATG start codon were cloned in a lentiviral packing vector (FhSynW2) containing the human synapsin promoter [34]. Poly-GA, poly-GR and poly-PR were expressed from synthetic genes devoid of GGGGCC repeats, while poly-GP was expressed from a ATG(GGGCCG)_80_ construct. For poly-GR, the GFP had to be fused to the N-terminus to allow robust expression (for details see [34]). Lentivirus was produced in HEK293FT cells (Life Technologies) as described previously [15].

### Cell culture

Primary hippocampal and cortical neurons were cultured from embryonic day 19 rats and infected for transduction with lentivirus as described previously [15, 48]. For immunofluorescence, the primary neurons were fixed for 10 min in 4 % paraformaldehyde and 4 % sucrose on ice. Primary and secondary antibodies were diluted in GDB buffer (0.1 % gelatin, 0.3 % Triton X-100, 450 mM NaCl, 16 mM sodium phosphate pH 7.4). Confocal images were taken by a LSM710 confocal laser scanning system (Carl Zeiss, Jena, Germany) with a 63× oil immersion objective.

### Patient material, brain slices

Tissue samples of all autopsy cases investigated were provided by the Neurobiobank Munich, Ludwig-Maximilians-University (LMU) Munich. They were collected according to the guidelines of the local ethical committee. Demographic and neuropathological data are listed in Table 1.

**Table 1:** Demographic and neuropathological data of patients and control cases

Case no.	Sex	Age at death	Duration of disease	Neuropathological diagnosis
C9-1	Female	65	3 years	FTLD–MND
C9-2	Female	59	6 months	FTLD–MND
C9-3	Male	65	4 years	FTLD–MND
C9-4	Female	63	3 years	MND
C9-5	Female	49	8 months	MND
C9-6	Male	51	2 years	MND
C9-7	Male	72	1 years	FTLD
C9-8	Female	57	7 years	FTLD
C9-9	Male	67	Unknown	FTLD
C9-10	Male	41	6 years	FTLD–MND
C9-11	Male	56	22 months	FTLD–MND
C9-12	Male	57	3 years	FTLD–MND
C9-13	Male	57	3–4 years	FTLD–MND
C9-14	Male	74	Several years	FTLD–MND
FUS-1	Female	54	4 years	FTLD–MND–FUS
Ctrl-1	Male	60	–	–
Ctrl-2	Female	60	–	–

### Definition of neuropathological groups

Cases with *C9orf72* mutation were stratified into frontotemporal lobar degeneration (FTLD), motoneuron disease (MND) or mixed FTLD/MND according to neuropathological criteria. FTLD was diagnosed when gliosis and/or spongy alterations were seen in the cortex of the superior and/or medial frontal gyrus (Brodman areas 8/9) and/or in the cortex of the parahippocampal and/or fusiform gyrus on hemalum–eosin stainings. MND was diagnosed when either the motor cortex showed gliosis and/or spongy alterations on hemalum–eosin stainings and/or the pyramidal tract showed a microglia activation on immunohistochemical stains using the CR3/43 antibody and/or the hypoglossal nucleus and/or the anterior horn at any spinal cord level showed a loss of motoneurons and/or gliosis and/or p62-positive inclusions in motoneurons.

### Immunohistochemistry

Immunohistochemistry and immunofluorescence were performed on paraffin sections as previously described [29]. For Unc119 immunohistochemistry, paraffin sections were treated 25 min with 0.1 µg/μl proteinase K in 10 mM Tris/HCl. This pretreatment dramatically increased the number of visible Unc119 aggregates (compare [34]). Afterwards the slides were incubated with the Unc119 antibody overnight at 4 °C and detected with the DCS SuperVision 2 Kit (DCS innovative diagnostic-system, Hamburg, Germany) according to the manufacturer’s instructions. An additional 0.05 µg/µl proteinase K pretreatment for 1 min before citrate retrieval was used for anti-nucleolin and H3K9me2 immunofluorescence experiments. Anti-poly-GA immunohistochemistry was performed with the Ventana BenchMark XT automated staining system (Ventana) using the UltraView Universal DAB Detection Kit (Roche). Incubation with poly-GR and poly-GP antibodies was done overnight at 4 °C, further steps were an incubation with a rabbit anti-rat antibody (1:2000) for 1 h at room temperature, and a final processing on the Ventana BenchMark XT using the UltraView Universal DAB Detection Kit (Roche). The poly-PR antibody was also incubated overnight at 4 °C and detected the following day on Ventata BenchMark XT. Images of immunohistochemical stainings were taken by CellD, Olympus BX50 Soft Imaging System (Olympus, Tokyo, Japan), confocal images on a LSM710 (Carl Zeiss) with a 40× or 63× oil immersion objective.

### RNA in situ hybridization

Paraffin sections were dewaxed in xylene and ethanol followed by microwaving in citrate pH6 buffer for 4 × 5 min. After washing with 2× SSC (0,3 M NaCl, 30 mM sodium citrate, pH7), sections were preincubated (30 min) at 65 °C in 2× SSC containing 40 % formamide and 2.5 % BSA and incubated over night at 50 °C with the Cy3(GGCCCC)_4_ probe (Integrated DNA Technologies, Coralville, Iowa) in 2× SSC containing 0.8 mg/ml tRNA, 0.8 mg/ml salmon sperm DNA, 0.16 % BSA, 8 % dextran sulfate, 1.6 mM ribonucleoside vanadyl complex and 5 mM EDTA. After washing with 0.5× SSC immunofluorescence was performed as described previously [29]. In all steps, RNase-free Milli-Q ultrapurified water was used.

### Semi-quantitative analysis of inclusion pathology

Frequency of poly-GA, poly-GR, poly-GP and Unc119 inclusion pathology was analyzed separately for neuronal cytoplasmic inclusions (NCI), neuronal intranuclear inclusions (NII) and dystrophic neurites (DN) in a semi-quantitative manner for 36 different CNS regions of five representative cases (C9-1 to 5) with *C9orf72* mutation with a Zeiss Axioplan microscope. In neocortical regions, in the granular and molecular cell layers of the cerebellum and in spinal cord, each type of inclusion pathology was considered as “few” if less than half of 12 representative visual fields (using a 20× objective) showed at least one inclusion, as “some” if more than half but not all visual fields showed at least one inclusion, as “many” if in every visual field at least 4 inclusions were detectable and as “abundant” if each visual field showed more than 20 aggregates. This method was also used for counting dystrophic neurites in all regions. In structures of hippocampus, subcortical nuclei, brain stem and the Purkinje cell layer of the cerebellum, NCIs and NIIs were considered as “few” if less than 2 % of the neurons contained aggregates, “some” if 3–25 % of the neurons contained aggregates, “many” if 25–50 % of the neurons contained aggregates and “abundant” if more than 50 % of the neurons contained aggregates.

### Quantitative analysis of inclusion pathology

The following areas with high loads of DPR protein aggregates but diverging neurodegenerative vulnerability were selected for quantification of NCIs and NIIs: cortex of the superior frontal gyrus, motor cortex, striate area of the occipital cortex, granular cell layer of the dentate gyrus, cornu ammonis regions 3/4, granular cell layer of the cerebellum, molecular cell layer of the cerebellum (superior part).

In all cases with *C9orf72* mutation, 3–12 pictures adjacent to each other were taken from a representative area of each region of interest with a digital camera (Olympus Cam SC30) at an Olympus BX41 microscope using a 40× objective for cerebellar granular cell layer and a 20× objective for all other regions. Three to four pictures were taken from each cerebellar and hippocampal region. In neocortex, pictures were taken in a columnar orientation covering all six cell layers. The inclusions of one such column represented by 6–12 adjacent pictures were counted. All NCIs and NIIs were manually counted on each digital picture separately using the CellCounter plugin in Fiji ImageJ. For each region in each case, the total number of inclusions was divided by the number of pictures taken, and the average value was determined. Finally, the average of the values for each region was determined in each neuropathological group (FTLD, MND, FTLD/MND) separately.

### Statistics

Statistical analysis was performed with GraphPad Prism software (version 6.01). The groups with neuropathological diagnosis MND, FTLD and FTLD/MND were compared and analyzed by two-way ANOVA followed by Tukey’s post hoc test. Nucleolus size (Feret diameter) was quantified from confocal images, taken on a LSM710 with a 40× oil immersion objective, using Fiji ImageJ particle analyzer and statistically evaluated by an unpaired *t* test followed by an *F*-test to compare variances. Multiple comparison of the size of the nucleoli in the frontal cortex was done by one-way ANOVA followed by Tukey’s post hoc test. Significance level was set at *p* < 0.05 (two sided).

## Results

### Intranuclear poly-GR and poly-PR inclusions are nucleolar in cell models, but para-nucleolar in patients

To compare DPRs expressed from synthetic genes and DPR inclusions in *C9orf72* mutation patients under optimal conditions, we raised novel monoclonal antibodies. Rat poly-GP antibody 7A5, rat poly-GR antibody 7H1 and mouse poly-PR antibody 32B3 specifically detected the respective 15-mer DPRs fused to GST (Fig. S1a). 7A5 and 7H1 robustly detected SDS-insoluble aggregates in frontal cortex of patients but not of controls cases (Fig. S1b). In patients, poly-GR antibody 7H1 detected more neuronal cytoplasmic inclusions than the previously used clone 5H9 (Fig. S1c). The monoclonal poly-GP and poly-PR antibodies also allowed a more sensitive detection of poly-GP and poly-PR inclusions than our previous polyclonal antibodies [36, 38]. With the new antibodies, poly-GR and poly-GP aggregates were found in various brain areas and in spinal cord motoneurons of *C9orf72* mutation patients, but not of control cases (Fig. S2a, b). Poly-PR inclusions were much less common in all brain regions (Fig. S2c). Despite a recent report of preferential aggregation of poly-PR in spinal cord motoneurons [8], we found no such inclusions with both the mouse poly-PR antibody 32B3 and our rabbit polyclonal antibody [39].

**Fig. 1:**
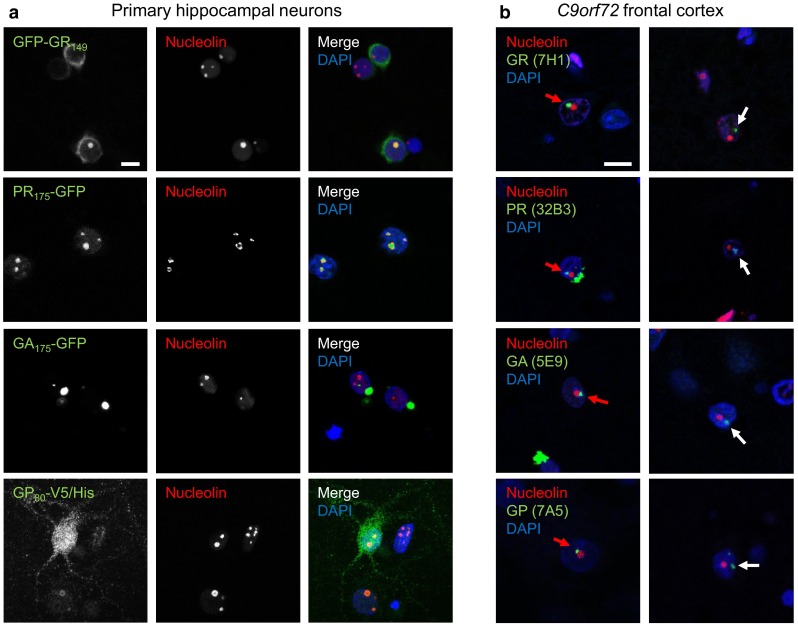
Differential localization of intranuclear DPR inclusions in transduced primary neurons and in neurons of cases with *C9orf72* mutation. Double immunofluorescence for different DPR proteins (*green*) and nucleolin (*red*), a marker for the nucleolus, in primary neurons (**a**) and in frontal cortex of cases with *C9orf72* mutation (**b**). Nuclei are labeled with DAPI. Single confocal sections containing the nucleolus are shown. **a** Primary neurons transduced with lentivirus expressing either GFP-GR_149_, PR_175_-GFP, GA_175_-GFP or GP_80_-V5/His (DIV6 + 7). Note that poly-GR and poly-PR but not poly-GA intranuclear inclusions are localized in the nucleolus. Poly-GA forms mainly compact cytoplasmic inclusions. Poly-GP expression is mainly pan-nuclear and also cytosolic. **b** In cortical areas of cases with *C9orf72* mutation neuronal intranuclear poly-GA, poly-GR and poly-GP inclusions are mostly localized adjacent to the nucleolus (*red arrows*) or less frequently randomly distributed (*white arrows*). No colocalization of DPR proteins with the nucleolus is observed. *Scale bars* represent 10 µm

To analyze the DPR proteins in vitro, we transduced rat hippocampal neurons with a lentivirus expressing GFP-GR_149_, PR_175_-GFP, GA_175_-GFP or GP_80_-V5/His for 7 days. Consistent with previous results [34, 57, 59], GFP-GR_149_ showed a diffuse cytoplasmic distribution and often formed nuclear aggregates that colocalized with nucleolin, a key component of the nucleolus (Fig. 1a, first row). PR_175_-GFP showed more pronounced nuclear and nucleolar localization and the majority of nucleoli appeared fragmented (Fig. 1a, second row). GA_175_-GFP formed compact mainly cytoplasmic and some intranuclear inclusions that did not colocalize with nucleolin (Fig. 1a, third row). GP_80_-V5/His expression was diffusely distributed throughout the neurons with some enrichment in the nucleus (Fig. 1a, fourth row). Lentiviral expression of the four DPR constructs in cortical neurons fully confirmed the localization found in hippocampal neurons (Fig. S3).

In contrast to transduced hippocampal neurons, poly-GR and poly-PR antibodies labeled mainly cytoplasmic inclusions in *C9orf72* mutation patients (Fig. S2a, c), an observation consistent with previous reports [17, 36, 38, 60]. However, a fraction of neurons also contained small poly-GR and poly-PR inclusions in the nucleus (Fig. 1b, first and second row). Quantitative analysis revealed that 78 % of the poly-GR NIIs were attached to the nucleoli, whereas the remaining NIIs were randomly distributed (Fig. 1b, first row, Fig. S4a). In contrast to GFP-GR_149_ and PR_175_-GFP expressing neurons, we never saw a colocalization of poly-GR or poly-PR and nucleolin in three *C9orf72* cases investigated. Immunofluorescence with two other monoclonal poly-GR antibodies (5H9 and 5A2) [36, 38] confirmed these results (Fig. S4b). Moreover, poly-GR did not colocalize with fibrillarin, another nucleolar marker (Fig. S4c). Intranuclear poly-GA and poly-GP showed a very similar pattern of para-nucleolar inclusions in *C9orf72* mutation patients (Fig. 1b, rows three and four; Fig. S4a). Thus, current cellular DPR models cannot fully replicate the pattern of intranuclear aggregates found in patient tissue.

### Para-nucleolar DPR aggregates colocalize with silent DNA

**Fig. 2:**
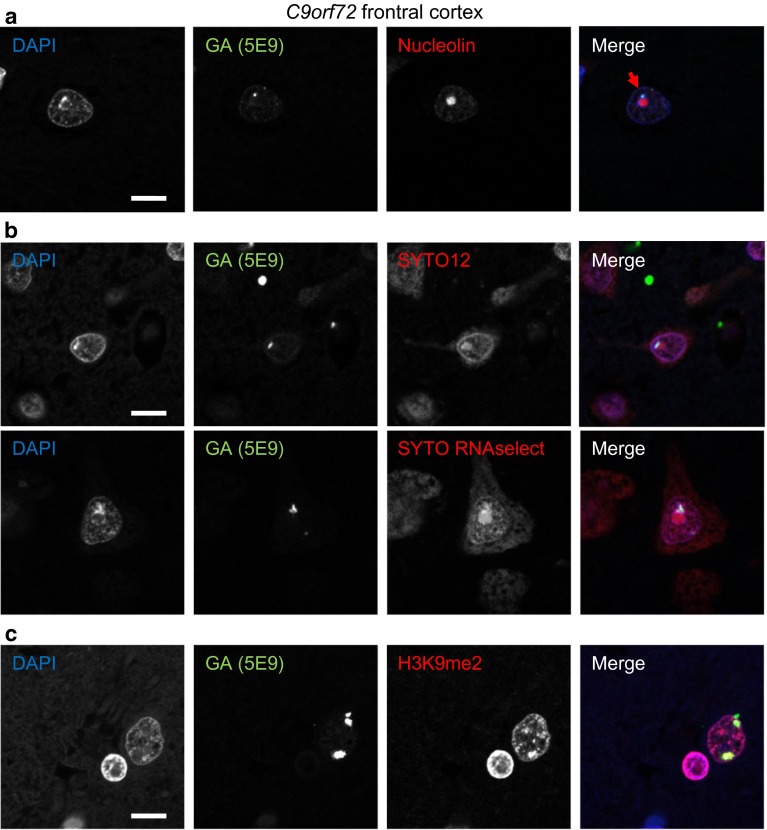
Para-nucleolar poly-GA inclusions colocalize with transcriptionally silenced DNA. Immunofluorescence for poly-GA proteins with the indicated antibodies and dyes to label DNA or RNA in frontal cortex of cases with *C9orf72* mutation. **a** Para-nucleolar poly-GA inclusions are enriched for heterochromatin labeled with the DNA-specific dye DAPI (*arrow*). **b** Para-nucleolar poly-GA inclusions are also stained with RNA-selective dyes SYTO12 and SYTO RNAselect. Note that both dyes also show chromatin staining similar to DAPI indicating cross-reactivity with DNA. **c** Nuclear poly-GA inclusions colocalize with histone 3 dimethylated at lysine 9 (H3K9me2), a marker for transcriptionally inactive DNA. *Scale bars* represent 10 µm

To elucidate the nature of the para-nucleolar DPR compartment, we analyzed colocalization with several marker proteins (data not shown). However, none of the markers for Marinesco bodies (HDAC6), the perinuclear compartment (CUG-BP1, PML, HSF1 and CD99), clastosomes (proteasomal subunits PSMC2 and PSMC4) and nucleolar caps (fibrillarin, coilin and PML) colocalized with para-nucleolar DPR inclusions, indicating they represent a unique compartment. Moreover, the para-nucleolar DPR protein aggregates were also not colocalized with the nuclear GGGGCC RNA foci in frontal cortex or cerebellum (Fig. S4d/e). However, many para-nucleolar DPR inclusions colocalized with heterochromatin detected by the DNA-binding dye DAPI in patients (Fig. 2a), which was not observed for poly-GA, poly-GR, poly-PR or poly-GP overexpressed in primary neurons (Fig. 1a). Para-nucleolar DPR inclusions were also labeled by the RNA-binding dyes SYTO12 and SYTO RNAselect, but no RNA enrichment was observed compared to the nucleolus (Fig. 2b). Since all RNA dyes also cross-react with DNA to some extent, we focused on the specific enrichment of heterochromatin DNA in para-nucleolar DPR inclusions. Colocalization was even more pronounced with an antibody for histone 3 dimethylated at lysine 9 (H3K9me2), a signal for transcriptional silencing (Fig. 2c). This may link para-nucleolar DPR proteins to transcriptional changes induced by the expanded *C9orf72* repeat DNA and RNA [20].

Since arginine-rich DPR proteins and transcription of the expanded repeat have been shown to induce nucleolar stress in cellular models [20, 50], we also investigated nucleolar size and morphology. Nucleolin stainings of the CA3/4 layer of the hippocampus, a region with abundant DPR pathology, revealed no differences in nucleolus shape and size between *C9orf72* patients and controls (Fig. S5a, b). In the frontal cortex of *C9orf72* FTLD cases, the size of the nucleoli did not differ from nucleoli of healthy controls regardless, whether the cells contained cytoplasmic or para-nucleolar or no DPR inclusions (Fig. S5c).

Nucleolar stress typically results in nucleolar p53 accumulation [26], which we did not observe in *C9orf72* cases (Fig. S5d). Thus, the expanded hexanucleotide repeat DNA and/or RNA may interfere with transcriptional processes without inducing overt nucleolar stress in the hippocampus and cortical areas.

### Overexpressed and patient poly-GR, poly-PR and poly-GP show different p62 labeling

**Fig. 3:**
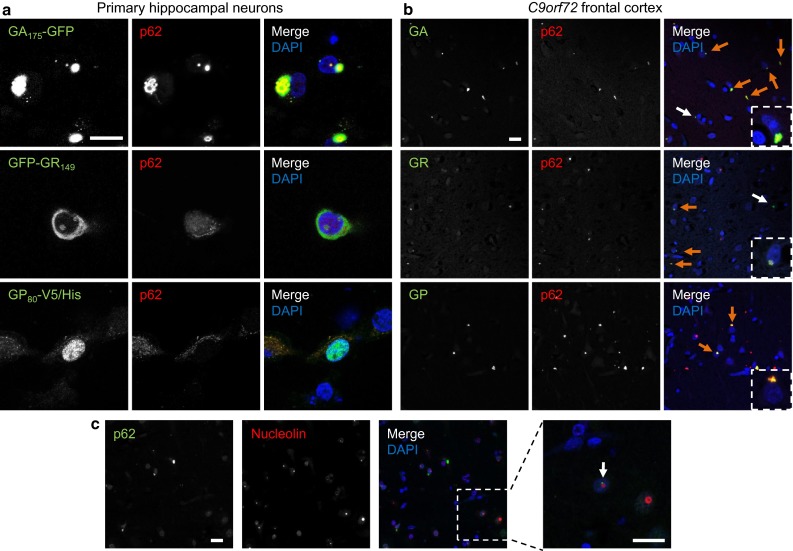
Differential colocalization of DPR and p62 inclusions in cases with *C9orf72* mutation and cell culture. Double immunofluorescence for DPR proteins and p62. Nuclei are labeled with DAPI. **a** In primary hippocampal neurons transduced with GA_175_-GFP, GFP-GR_149_ or GP_80_-V5/His (DIV6 + 7) p62 co-aggregates with cytoplasmic and intranuclear poly-GA inclusions, but not with poly-GR and poly-GP. In contrast to poly-GA inclusions, poly-GR and poly-GP aggregates appear less compact or granular. **b** In frontal cortex of *C9orf72* mutation patients almost all poly-GA and poly-GR and all poly-GP inclusions are positive for p62 (*orange arrows*). Poly-GA and poly-GR inclusions without p62 labeling are rare (*white arrows*). **c** Intranuclear p62 aggregates show the same distribution pattern as DPR inclusions and are mostly para-nucleolar (*arrow*). *Scale bars* represent 20 µm

p62 is found in many inclusion bodies of neurodegenerative diseases. Although most inclusions of all DPR species colocalize with p62 in *C9orf72* patients [36, 38], we and others had only found a colocalization of p62 with overexpressed poly-GA but not with other overexpressed DPR species in HEK293 cells [34, 57]. We therefore tested p62 co-aggregation in primary hippocampal neurons with lentiviral expression of GA_175_-GFP, GFP-GR_149_, PR_175_-GFP, GP_80_-V5/His. Consistent with previous results, most cytoplasmic and intranuclear GA_175_-GFP inclusions were strongly co-labeled with p62 antibodies (Fig. 3a, first row), while GFP-GR_149_ and PR_175_-GFP inclusions were negative for p62 (Fig. 3a, second row and Fig. S6a). GP_80_-V5/His was diffusely expressed with enrichment in the nucleus without obvious p62 colocalization (Fig. 3a, third row). These results were confirmed in cortical neurons transduced with the same DPR constructs (Fig. S6b).

We wondered whether such p62-negative poly-GR inclusions occur in patients, particularly in the nucleolus. In frontal cortex, double immunostaining revealed a strong colocalization of poly-GR and p62 in the cytosol and the nucleus, similar to poly-GA (Fig. 3b, first and second row, Fig. S7a, first row). Only very few poly-GR inclusions in the cytosol (Fig. 3b, second row) as well as in the nucleus (Fig. S7a, second row) were not labeled with p62. Similarly, the vast majority of poly-GP and poly-PR inclusions co-stained with p62 (Fig. 3b, third row and Fig. S7b).

Moreover, double immunostaining of p62 and nucleolin revealed no colocalization of ubiquitinated inclusions and the nucleolus (Fig. 3c). However, occasionally p62 labeling was observed next to the nucleolus, which was consistent with the findings for specific DPR antibodies (Fig. 1b). Together, these findings indicate that in patients with *C9orf72* mutation most intranuclear DPRs aggregate in a p62-positive para-nucleolar compartment and not directly within the nucleolus.

### Poly-GR and poly-GP inclusion types resemble poly-GA pathology and also occur in glia

**Fig. 4:**
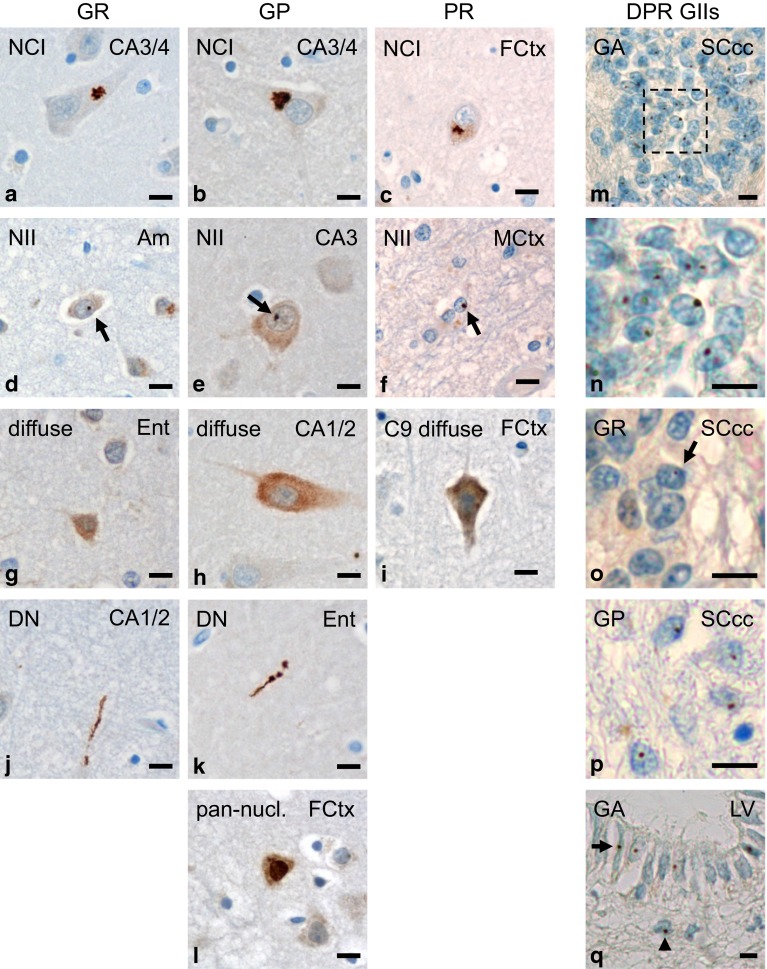
Spectrum of DPR pathology in neurons and glial cells of patients with *C9orf72* mutation. **a–l** Immunohistochemistry with novel monoclonal antibodies for poly-GR (clone 7H1), poly-GP (clone 7A5) and poly-PR (clone 32B3) in cases with *C9orf72* mutation. Poly-GR, poly-GP and poly-PR mainly form compact characteristic star-like cytoplasmic (**a–c**) or small round intranuclear inclusions (*arrows* in **d**–**f**) in neurons and show a diffuse granular cytoplasmic staining (**g**–**i**). Furthermore there are poly-GR and poly-GP aggregates in dystrophic neurites (**j**, **k**); note that dystrophic neurites with poly-PR could not be detected. Solely for poly-GP, a diffuse pan-nuclear staining is also found (**l**). **m–q** Immunohistochemistry with indicated DPR antibodies shows glial intranuclear inclusions in *C9orf72* cases. In ependymal cells of spinal cord central canal intranuclear inclusions of poly-GA (**m**, **n** detail of **m**), and less frequently of poly-GR (**o**) and poly-GP (**p**) are detectable; further glial intranuclear inclusions of poly-GA are present in ependymal (*arrow*) and subependymal (*arrowhead*) cells of the lateral ventricle wall at level of accumbens nucleus (**q**). *Scale bars* represent 20 µm. *Am* amygdala, *CA* cornu ammonis region, *DN* dystrophic neurite, *Ent* entorhinal cortex, *FCtx* frontal cortex, *GII* glial intranuclear inclusion, *MCtx* primary motor cortex, *LV* wall of lateral ventricle, *NCI* neuronal cytoplasmic inclusion, *NII* neuronal intranuclear inclusion, *SC* spinal cord, *SCcc* spinal cord central canal

To further analyze the correlation of DPR inclusions with neurodegeneration, we characterized the spectrum of poly-GR, poly-GP and poly-PR pathology in *C9orf72* mutation patients. Poly-GR (7H1), poly-GP (7A5) and poly-PR (32B3) antibodies labeled predominantly NCIs throughout the brain, which showed the characteristic star-shaped appearance in pyramidal cells of the hippocampal formation and cortical neurons (Fig. 4a–c). Additionally, NIIs and “pre-inclusions” with diffuse cytoplasmic staining were also detected with all three DPR antibodies (Fig. 4d–i). Only poly-GR and poly-GP antibodies also detected DNs (Fig. 4j, k). Additionally, poly-GP antibodies occasionally visualized diffuse pan-nuclear DPR expression (Fig. 4l), resembling the pattern of recombinant poly-GP expression in neurons (Figs. 1a, 3a, S3).

Although DPR proteins had previously been described exclusively in neurons, we noticed intranuclear inclusions in ependymal cells of the spinal cord central canal in *C9orf72* cases with MND most prominently with poly-GA antibodies (Fig. 4m, n), but also with poly-GR and poly-GP antibodies (Fig. 4o, p). Such glial inclusions were not detected in an FTLD–MND–FUS case confirming antibody specificity (Fig. S7c). Strikingly, the vast majority of these inclusions were intranuclear, while most neuronal DPR inclusions were cytoplasmic. In contrast to neuronal intranuclear DPR inclusions, the ependymal inclusions were not associated to the nucleolus (Fig. S7d). We observed further glial intranuclear poly-GA inclusions in ependymal and subependymal cells lining the lateral ventricle (Fig. 4q). Thus, not only TDP-43 pathology but also DPR pathology extends to glial cells in *C9orf72* mutation patients.

Taken together, the poly-GR and poly-GP inclusion pattern resembled that of poly-GA in *C9orf72* mutation patients [10, 29, 38]. Poly-PR inclusions were very rare and were not found in DNs. The identification of different types of inclusions in neuronal and glial cells suggests cell type-dependent differences in DPR aggregation or degradation.

### Spectrum and distribution of DPR inclusions

To further elucidate the spectrum of DPR pathology in *C9orf72* mutation patients, we analyzed the load of NCI, NII and DN pathology in 36 CNS regions using monoclonal antibodies for poly-GA (clone 5E9), poly-GR (clone 7H1) and poly-GP (clone 7A5) in five representative cases with comprehensive tissue collection, including two MND cases and three FTLD/MND cases (C9-1 to C9-5, see Table 1). Overall poly-PR distribution pattern appeared similar (not shown), but the number of inclusions was too low for a reliable semi-quantitative analysis.

**Fig. 5:**
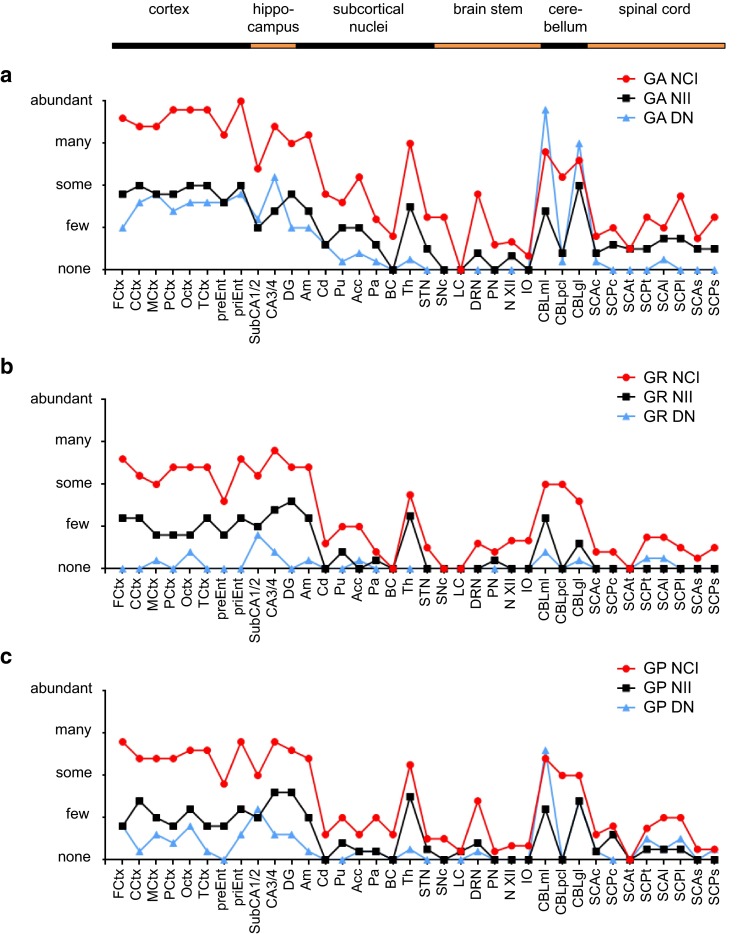
Regional distribution pattern of neuronal poly-GA, poly-GR and poly-GP inclusion pathology in cases with *C9orf72* mutation. Semi-quantitative immunohistochemical analyses for poly-GA (**a**), poly-GR (**b**) and poly-GP (**c**) neuronal cytoplasmic inclusions (NCI), neuronal intranuclear inclusions (NII) and dystrophic neurites (DN) in representative cortical, hippocampal, subcortical, brain stem, cerebellar and spinal cord areas of five *C9orf72* mutation patients reveal a predominance of poly-GA aggregates for all types of aggregates in all areas. Highest densities of poly-GA, poly-GR and poly-GP aggregates are seen in cortical areas, hippocampus, amygdaloid nuclei, thalamus and cerebellum. Note that poly-GR-positive DNs are rarely found outside the hippocampus. Categories for semi-quantitative analysis (none, few, some, many, abundant) are explained in detail in the “Materials and methods” section. *Acc* accumbens nucleus, *Am* amygdaloid nuclei, *BC* basal nucleus of Meynert compact part, *CA3/4* cornu ammonis fields 3/4, *CBLgl* cerebellar granular cell layer, *CBLml* cerebellar molecular cell layer, *CBLpcl* cerebellar Purkinje cell layer, *CCtx* cortex of cingulate gyrus, *Cd* caudate nucleus, *DG* dentate gyrus, *DRN* dorsal raphe nuclei, *FCtx* frontal cortex, *IO* inferior olive, *LC* locus coeruleus, *MCtx* primary motor cortex, *N XII* hypoglossal nucleus, *OCtx* occipital cortex, *Pa* pallidum, *PCtx* parietal cortex, *PN* pontine nuclei of pons, *preEnt* lamina principalis externa of entorhinal cortex, *priEnt* lamina principalis interna of entorhinal cortex, *Pu* putamen, *SCAc* anterior horn of cervical spinal cord, *SCAl* anterior horn of lumbar spinal cord, *SCAs* anterior horn of sacral spinal cord, *SCAt* anterior horn of thoracic spinal cord, *SCPc* posterior horn of cervical spinal cord, *SCPl* anterior horn of lumbar spinal cord, *SCPs* posterior horn of sacral spinal cord, *SCPt* posterior horn of thoracic spinal cord, *SNc* substantia nigra compact part, *STN* subthalamic nucleus, *SubCA1/2* subiculum plus cornu ammonis fields 1/2, *TCtx* temporal cortex, *Th* thalamus

In all brain regions, DPR inclusion pathology in form of NCIs, NIIs and DNs was most abundant for poly-GA (Fig. 5a) and less distinct for poly-GR and poly-GP (Fig. 5b, c; Table S1). Regardless of the neuropathological diagnosis, all cases showed the strongest DPR pathology in neocortex, hippocampus and cerebellum. DPR inclusions were also abundant in amygdala and thalamus. Few inclusions were visible in basal ganglia, brain stem and spinal cord. Overall, DNs with poly-GR aggregates were less frequent than those with poly-GA or poly-GP aggregates. The highest density of poly-GA or poly-GP containing DNs was seen in the molecular layer of the cerebellum. Despite the abundant intranuclear inclusions of overexpressed poly-GR in various cell models, poly-GR NIIs were even less frequent than poly-GA and poly-GP NIIs in *C9orf72* mutation patients. Poly-GR NIIs were most abundant in the thalamus compared to poly-GR NCIs. Thus, the pattern of poly-GA, poly-GR and poly-GP inclusions pathology is consistent with previous less detailed reports [1, 29, 36]. The biggest difference between the three sense strand-derived DPR species was the almost complete lack of poly-GR DNs throughout the CNS.

### Poly-PR but not poly-GR inclusions show different distribution in FTLD and MND cases

**Fig. 6:**
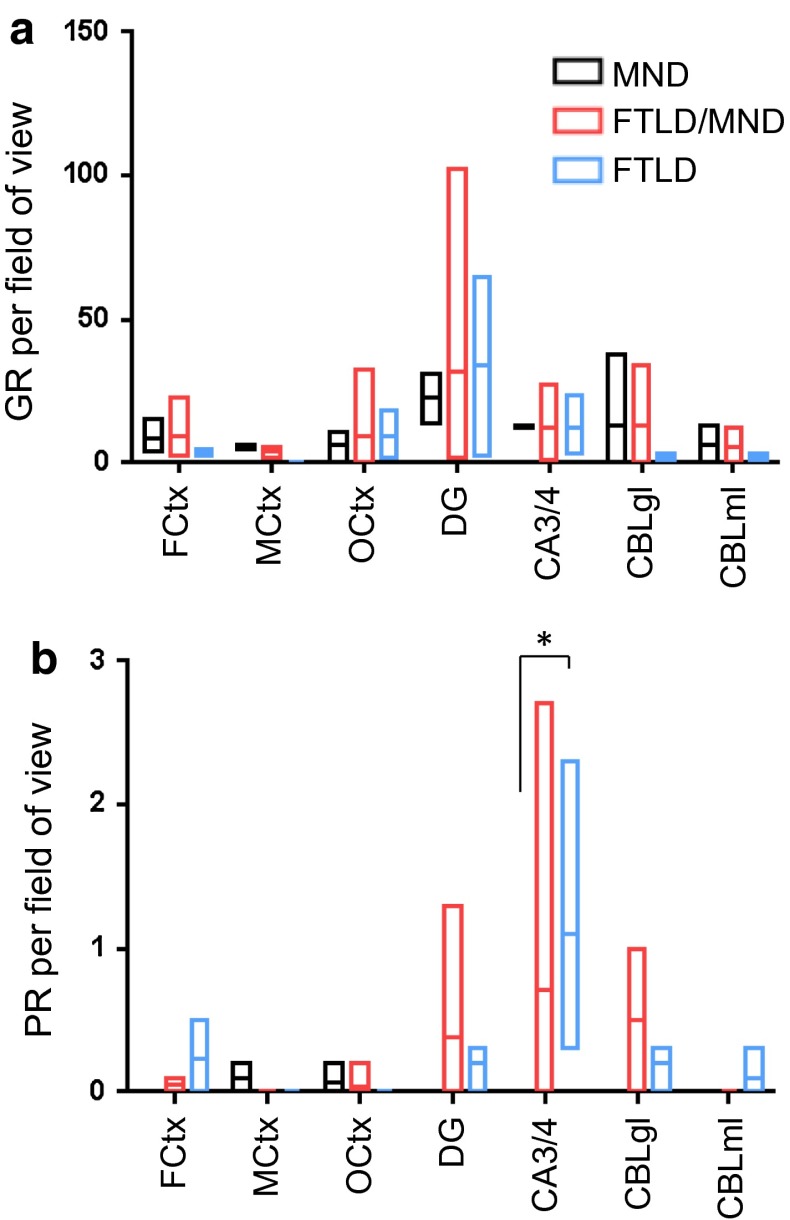
Quantitative assessment of poly-GR and poly-PR inclusion pathology in selected brain areas of *C9orf72* cases with different neuropathological phenotypes. The graphs show the minimum, mean and maximum number of poly-GR and poly-PR inclusions averaged per visual field. **a** There is no significant difference in the average number of cytoplasmic and intranuclear neuronal poly-GR inclusions per visual field (20× objective, 40× for cerebellar granular cell layer) between *C9orf72* cases with motoneuron disease (MND, *n* = 2–3), cases with frontotemporal lobar degeneration (FTLD, *n* = 3, no MtCtx) and cases with a combination of frontotemporal lobar degeneration and motoneuron disease (FTLD/MND, *n* = 4–8) in brain areas with highest poly-GR load. **b** Poly-PR inclusions are only common in hippocampus and cerebellum of FTLD and FTLD/MND cases, but absent in MND cases, reaching statistical significance in CA3/CA4 (ANOVA, *p* = 0.0103). The quantitative analysis is explained in detail in the “Materials and methods” section. The data for individual cases are presented in Table S2. *CA3/4* cornu ammonis fields 3/4, *CBLgl* cerebellar granular cell layer, *CBLml* cerebellar molecular cell layer, *DG* dentate gyrus, *FCtx* frontal cortex, *MCtx* primary motor cortex, *OCtx* occipital cortex

To better analyze the correlation of poly-GR and poly-PR pathology with neurodegeneration, we focused on seven key regions that are variably affected in *C9orf72* mutation patients. We counted the number of inclusions in a defined number of visual fields in three neocortical regions (cortex of the medial frontal gyrus, motor cortex striate area of the occipital cortex), two hippocampal regions (granular cell layer of the dentate gyrus, pyramidal cell layer of cornu ammonis regions 3 and 4) and the granular and molecular cell layers of the cerebellar cortex (for details see methods). Compared to the semi-quantitative analysis (Fig. 5), we used a larger cohort of 14 patients, including three MND cases, three FTLD cases and eight patients with combined FTLD/MND (Table 1). Strikingly, poly-GR load was similar in occipital cortex, which is not affected by neurodegeneration in any of the three patient groups, and in frontal cortex, which is degenerated in FTLD and FTLD/MND cases, but not in MND cases (Fig. 6a; Table S2). In contrast, DPR abundance was less in the motor cortex than in frontal or occipital cortex, although we did not have material for comparison from patients without neuropathological signs of MND. Overall, poly-GR inclusions showed a very similar distribution pattern among all three patient subgroups, suggesting that poly-GR aggregation does not spatially correlate with neurodegeneration in *C9orf72* mutation patients.

Poly-PR inclusions were scarce throughout the CNS with the highest frequency in the hippocampus. In three cases (MND and FTLD/MND, 6 sections each), we found no poly-PR in spinal cord motoneurons. Poly-PR was significantly more abundant in the CA3/4 region of FTLD cases compared to MND cases (Fig. 6b). Thus, poly-PR, but not poly-GR, distribution differs between *C9orf72* disease subtypes, although it is not spatially correlated with neurodegeneration.

### Spectrum of Unc119 inclusion pathology

**Fig. 7:**
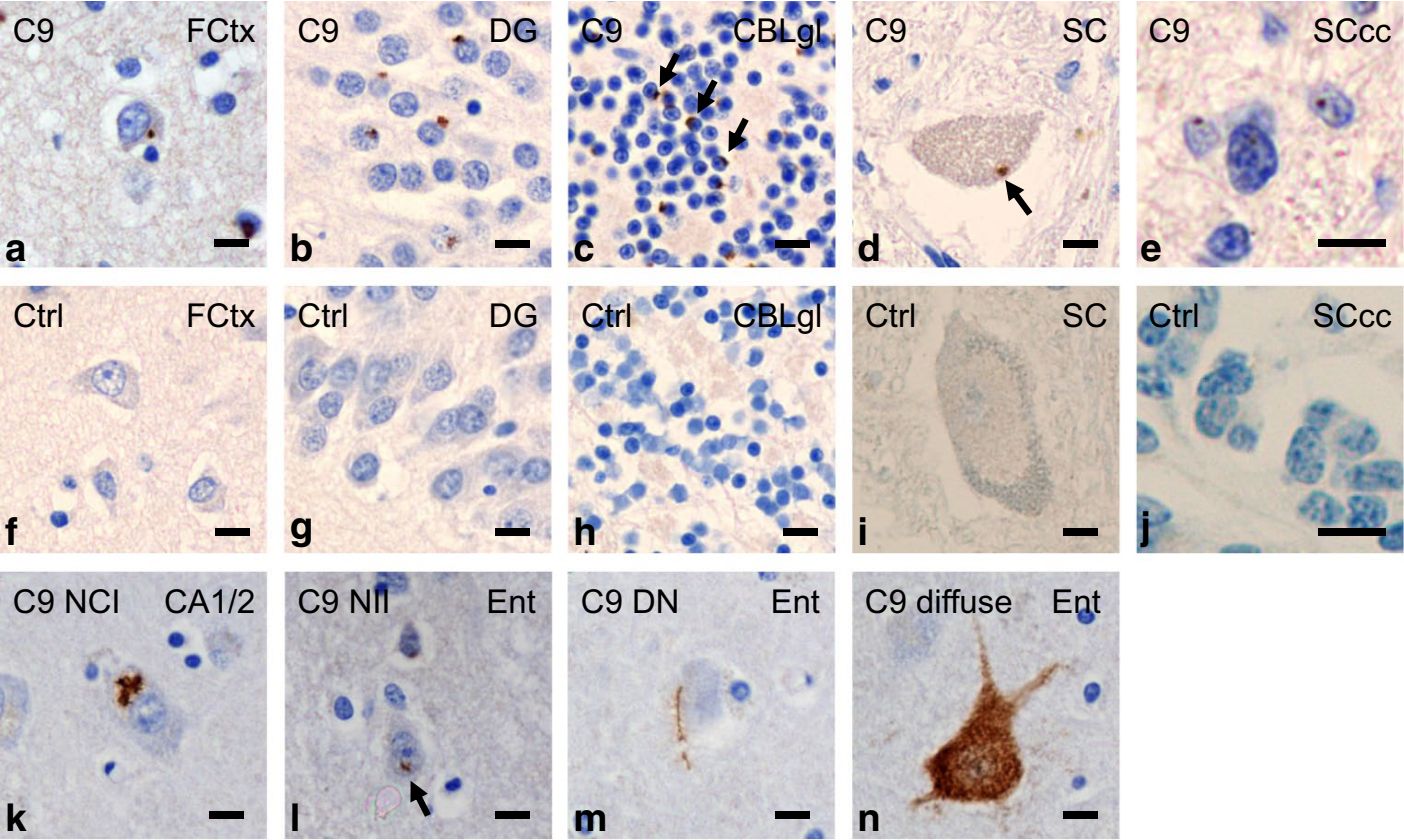
Spectrum of Unc119 pathology in cases with *C9orf72* mutation resembles poly-GA pathology. Immunohistochemistry with a polyclonal rabbit antibody against Unc119. In *C9orf72* mutation cases, numerous Unc119 inclusions are seen in neurons of various brain areas (**a–c**), rarely in motoneurons of spinal cord (**d**) and in ependymal cells of spinal cord central canal (**e**); examples of cytoplasmic inclusions are marked by *arrows*. (**f–j**) Corresponding areas of control cases do not contain such inclusions and the proteinase K pretreatment removes all soluble Unc119 staining in cases and controls. (**k–n**) The types of Unc119 aggregates are similar to those of DPR proteins. There are often star-like neuronal cytoplasmic inclusions (NCI) (**k**), neuronal intranuclear inclusions (NII, pointed by *arrow* in **l**), compact aggregates in dystrophic neurites (DN) (**m**) and diffuse granular cytoplasmic aggregates in neurons (**n**). *Scale bars* represent 20 µm. *C9* case with *C9orf72* mutation, *CA1/2* cornu ammonis fields 1/2, *CBLgl* granular cell layer of cerebellum, *Ctrl* control case, *DG* dentate gyrus, *Ent* entorhinal cortex, *FCtx* frontal cortex, *SC* spinal cord, *SCcc* spinal cord central canal

Next, we analyzed the distribution of Unc119, a transport factor for myristoylated proteins, which co-aggregates with poly-GA [34]. In our previous analyses, Unc119 inclusions were more prominent in regions affected by prominent neurodegeneration in three *C9orf72* mutation patients, but staining intensity and inclusions density varied considerably between patients. To improve detection of Unc119 inclusions, we tested several conditions for antigen retrieval (see method section for details). Brief proteinase K treatment completely removed the diffuse Unc119 staining in the neuronal soma of patients and controls, but dramatically increased visible Unc119 inclusion pathology in *C9orf72* mutation patients (Fig. 7). Using this improved staining protocol, we identified abundant Unc119 inclusions not only in the frontal cortex, the dentate gyrus but also in the cerebellum (Fig. 7a–c). Rare Unc119 inclusions were also seen in the cytoplasm of spinal cord motoneurons (Fig. 7d) and in the nuclei of central canal ependymal cells (Fig. 7e). No Unc119 inclusions were found in control cases (Fig. 7f–j). The spectrum of proteinase K resistant Unc119 pathology ranged from predominant NCIs to less abundant NIIs and DNs and to rare diffuse aggregates (Fig. 7k–n). Moreover, para-nucleolar Unc119 inclusions colocalizing with poly-GA were found, indicating that Unc119 can be recruited into the nucleus by poly-GA aggregates (Fig. S8). Overall, the pattern of Unc119 pathology in cases with *C9orf72* mutation strongly resembled the pattern of DPR pathology.

### Regional distribution of poly-GA and Unc119 inclusions differs between MND and FTLD cases

**Fig. 8:**
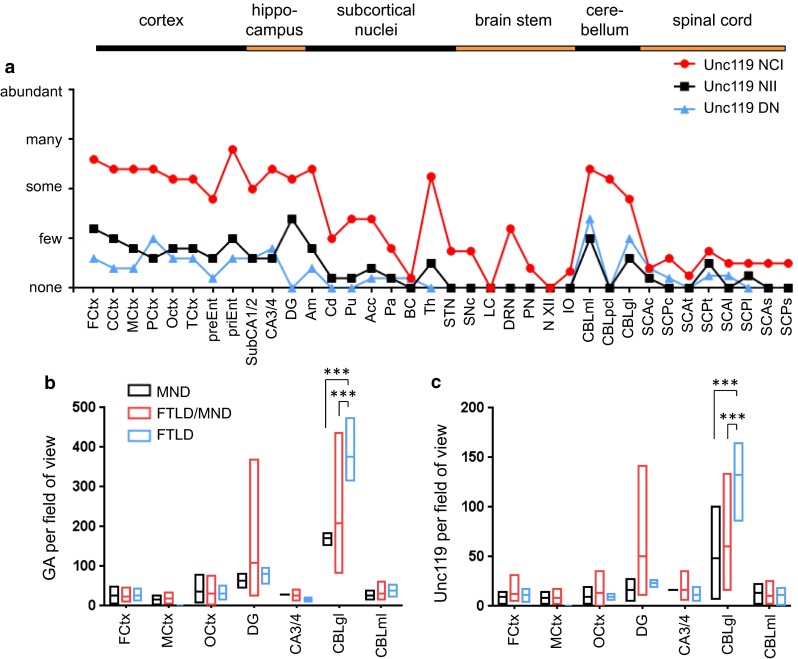
Distribution of poly-GA and Unc119 inclusion pathology depends on pathological subtypes. **a** Semi-quantitative analysis of Unc119 neuronal cytoplasmic inclusions (NCI), neuronal intranuclear inclusions (NII) and dystrophic neurites (DN) in representative cortical, hippocampal, subcortical, brain stem, cerebellar and spinal cord areas of five *C9orf72* mutation patients. The regional distribution of Unc119 inclusions resembles the pattern of poly-GA pathology (Fig. 5), albeit at overall lower abundance. Semi-quantitative analysis is explained in detail in the “Materials and methods” section. Abbreviations as in Fig. 5. **b**, **c** Quantitative analysis of NCI and NII of poly-GA and Unc119 pathology by immunohistochemistry in *C9orf72* mutation patients with MND (*n* = 2–3), FTLD (*n* = 3, no MtCtx) and combined FTLD/MND (*n* = 4–8) cases as in Fig. 6. The graphs show the minimum, mean and maximum number of poly-GA and Unc119 inclusions averaged per visual field. Poly-GA distributions are significantly different between FTLD, FTLD/MND and MND patients in granular layer of cerebellum (p_(FTLD vs. FTLD/MND)_ = 0.0003, p_(FTLD vs. MND)_ = 0.0003) (**b**). Similarly, the frequency of Unc119 inclusions is different in the granular layer of cerebellum in FTLD patients compared with FTLD/MND and MND (p_(FTLD vs. FTLD/MND)_ = 0.0005, p_(FTLD vs. MND)_ = 0.0008) (**c**). Quantitative analysis is explained in detail in the “Materials and methods” section, the data for individual cases are presented in Table S2. Abbreviations as in Fig. 6

To analyze the correlation of Unc119 aggregation and neurodegeneration, we extended our analysis to further CNS regions in the five representative cases (C9-1 to C9-5, see Table 1). We found many Unc119 inclusions throughout the neocortex, hippocampus and thalamus (Fig. 8a; Table S1). In contrast to findings using our previous staining protocol, Unc119 inclusions were now also frequent in the cerebellum. Overall, Unc119 distribution closely resembled poly-GA distribution (Fig. 5a), although Unc119 inclusions were less frequent in all brain regions (Fig. 8a). Unc119 NIIs were most prominent in the dentate gyrus and completely absent in the brain stem.

A quantitative analysis of the complete patient cohort revealed no difference in the poly-GA and Unc119 frequency in cortical regions and hippocampus between the MND, FTLD or FTLD/MND patients (Fig. 8b, c; Table S2). As for poly-GR (Fig. 6a), the poly-GA load was similar in the non-degenerating occipital cortex and the degenerating frontal cortex of FTLD and FTLD/MND patients (Fig. 8b). Unexpectedly, poly-GA and Unc119 inclusions were significantly more common in the cerebellar granular cell layer of FTLD patients compared to MND or FTLD/MND patients (Fig. 8b, c). Interestingly, these patients showed a trend towards lower levels of poly-GR inclusions (Fig. 6a), suggesting differential translation or aggregation of these DPR species in the cerebellum. These findings are consistent with an emerging role of the cerebellum in the pathophysiology of *C9orf72* disease [13, 14, 30–32, 52, 56].

## Discussion

With this study, we provide the first quantitative analysis of the three major DPR species poly-GA, poly-GR and poly-GP as well as poly-PR in a neuropathologically characterized cohort of *C9orf72* mutation patients using monoclonal antibodies. Despite ample in vitro evidence especially for poly-GA, poly-GR and poly-PR toxicity [23, 34, 35, 50, 55, 57, 59, 60], we could not identify a spatial correlation between DPR inclusion pathology and neurodegeneration in patients, although poly-GA and poly-PR showed different distribution in MND and FTLD cases. Different localization and aggregation behavior especially of poly-GR and poly-PR proteins in cellular models and patients may explain the poor translatability of the in vitro results. The newly identified para-nucleolar aggregation of DPR proteins in heterochromatin structures in patient neurons hints for repeat-associated alterations in transcription.

### Subcellular localization of DPR proteins

In patients, poly-GA, poly-GR, poly-GP and poly-PR showed remarkably similar regional and subcellular expression patterns, suggesting that these proteins are co-translated in most cells and then co-aggregate in p62-positive inclusions [38]. In transduced primary neurons, only poly-GA expression gives rise to p62-positive compact cytoplasmic inclusions. Consistent with previous reports overexpressed poly-GR and poly-PR predominantly localized to the nucleolus and was p62 negative in primary neuron culture [34, 50, 57]. However, in patients with *C9orf72* mutation, poly-GR and poly-PR inclusions were predominantly cytoplasmic, and we did not find a single nucleolar inclusion. Overexpression of poly-GP in neurons resulted either in diffuse cytoplasmic or more often diffuse pan-nuclear accumulation similar to previous reports [57]. We found both expression patterns in patients, although compact NCIs were much more common.

We noticed abundant poly-GA pathology in ependymal cells of the spinal cord central canal and the lateral ventricle. Poly-GP and poly-GR were detected at a lesser extent. While TDP-43 pathology and RNA foci have been detected in neurons and glia [17], DPR proteins had been described only in neurons and in Sertoli cells of testis so far [1, 38]. In contrast to neurons, ependymal cells harbor almost exclusively intranuclear inclusions. The pathogenic role of glial DPR inclusions remains unclear, since it does not extend to astrocytes and oligodendrocytes [29]. However, trophic support from ependymal cells has been linked to ALS either directly or via altering neurogenesis [6, 12]. Moreover, poly-GP has been detected in the CSF, which may reflect neuronal death or active secretion [47]. Additionally, ependymal cells may release DPR proteins into the CSF more efficiently than neurons.

Since the localization of DPR aggregates is already differing between neurons and glia in patients, cell type-specific effects may contribute to the aberrant expression pattern of overexpressed poly-GR and poly-GP in cellular models. Further explanations may be the faster expression kinetics and the lack of expression of the other DPR species and hexanucleotide repeat RNA in most current model systems. Since aberrantly localized DPR proteins may invoke different toxic pathways, future studies of cellular and animal models of DPR toxicity will benefit from the careful analysis of the subcellular localization of the aggregates.

### Para-nucleolar DPR aggregates and nucleolar stress

While intranuclear DPR inclusions appear randomly distributed throughout the nucleus in glia, we noticed that intranuclear inclusions in neurons are predominantly para-nucleolar. To elucidate the function of para-nucleolar DPR aggregates, we tested several markers for known nucleolus-associated compartments. Robust co-staining with p62 is reminiscent of the ubiquitinated Marinesco bodies, found in the aging brain particularly in neuromelanin containing neurons of the substantia nigra [3, 40]. However, the para-nucleolar DPR inclusions lack the characteristic eosinophilic staining and we could not detect colocalization with HDAC6, which had previously been identified in Marinesco bodies [40]. The “perinucleolar compartment” has been implicated in RNA polymerase III-dependent transcription [41], but the marker proteins CUG-BP1, PML, HSF1 and CD99 did not colocalize with DPR NIIs. In cells with elevated proteasomal activity, proteasomes congregate in “clastosomes” close to the nucleolus [24], but the para-nucleolar DPRs were negative for proteasomal subunits PSMC2 and PSMC4. Block of transcription leads to a segregation of nucleolar subcompartments and formation of the so-called “nucleolar caps”, but the marker proteins fibrillarin, PML and coilin were not detected in DPR inclusions [45].

Colocalization of para-nucleolar DPR proteins with heterochromatin in DAPI staining and with H3K9me2, a prominent marker of transcriptional repression, suggests a link between DPRs and transcriptional regulation. This is most consistent with transcriptional stalling and nucleolar stress due to formation of RNA·DNA hybrids (so-called R-loops) from hexanucleotide repeats [20]. Importantly, H3K9 dimethylation has been linked to R-loop-induced transcriptional silencing [46]. This potential link of DPR proteins with DNA/RNA-based disease mechanisms may also explain why para-nucleolar DPR aggregates were not found in transduced neurons expressing DPR proteins from synthetic genes. We found no colocalization of para-nucleolar DPR inclusions with GGGGCC repeat RNA foci. Consistent with previous reports, there was rather an inverse correlation of foci and (cytoplasmic) DPR inclusions [17]. Nucleolar stress is typically associated with nucleolar enlargement and nucleolar accumulation of p53 particularly when it is caused by proteasomal inhibition [21, 26]. Interestingly, two groups reported proteasomal impairment by poly-GA in vitro [57, 59]. However, we detected no nucleolar accumulation of p53 and no change in nucleolar size and morphology in *C9orf72* patients. Thus, neither the *C9orf72* mutant allele nor cytoplasmic or para-nucleolar DPR inclusions affected nucleolar size in the brain.

### Correlation of DPR and Unc119 inclusion pathology with neuropathological subtypes

Our cohort of 14 *C9orf72* mutation patients represents the whole spectrum of clinical and neuropathological subtypes, including three cases each with either MND or FTLD and eight cases with a mixed disease. We chose five representative cases with comprehensive tissue collection for the semi-quantitative analysis of 36 CNS regions of the sense strand-derived DPR species and Unc119. We had previously shown that poly-GA sequesters Unc119, a protein that regulates trafficking of lipidated cargo proteins, such as transducin α in the retina [34, 58]. Loss of Unc119 is neurotoxic and Unc119 overexpression rescues poly-GA toxicity in vitro. Using improved antigen retrieval with proteinase K, we could detect Unc119 in about 40 % of poly-GA inclusions in all analyzed brain regions. Although these data corroborate Unc119 as a specific component of poly-GA inclusions, selective co-aggregation of Unc119 cannot easily explain selective vulnerability in certain brain regions. However, proteinase K pretreatment precludes analyzing the residual soluble Unc119 in affected cells. Identification of Unc119 cargos essential for neuronal survival and analysis of their localization in *C9orf72* patients will be necessary to determine functional Unc119 inactivation and its correlation to neurodegeneration.

In all patients, DPR and Unc119 pathology showed a stereotypic expression pattern with highest abundance in cortex, hippocampus, thalamus and cerebellum. In contrast to previous semi-quantitative studies restricted to poly-GA pathology [10, 29], we performed a quantitative analysis of poly-GA, poly-GR, poly-PR and Unc119 pathology in seven critical brain regions in all 14 patients. The amount of poly-GA, poly-GR, poly-PR and Unc119 aggregates was similar in frontal cortex, motor cortex and occipital cortex, although the latter is not affected by neurodegeneration in *C9orf72* mutation patients. Moreover, the extent of DPR pathology in frontal cortex and motor cortex did not correlate with neurodegeneration in FTLD or MND cases. Interestingly, poly-GA and poly-PR, the DPR species with the strongest toxic effects in cell culture, showed distinct depositions in FTLD vs. MND cases with *C9orf72* mutation cases [34, 35, 55, 57, 59]. Poly-PR aggregates were significantly more common in the CA3/4 region of FTLD than of MND cases. Due to the very low frequency of poly-PR inclusions, the pathophysiological relevance remains unclear. Interestingly, nuclear foci of antisense repeat RNA have recently been linked to motor neuron degeneration [8]. Poly-GA and Unc119 pathology was significantly higher in the cerebellar granular cell layer of FTLD patients compared to MND and FTLD/MND patients. At the same time, there was a trend for lower poly-GR pathology in FTLD patients, which suggests that the composition of the DPR inclusions in these patients is significantly altered, although it is unclear if and how this is related to pathogenesis. In our previous study, focusing on poly-GA pathology no similar correlation was observed [29], but both studies differ in staging of the cases (clinically vs. neuropathologically) and in analyzing the extent of DPR pathology (semi-quantitative vs. quantitative approaches). Interestingly, there is considerable somatic heterogeneity in the length of the expanded *C9orf72* repeat and only the repeat length in the cerebellum but not in the frontal cortex is inversely correlated with disease duration, arguing for an underappreciated role of the cerebellum in the pathogenesis of FTLD [49, 52].

Overall, our data do not support a spatial correlation of DPR inclusions with neurodegeneration, although DPR proteins can clearly induce neurotoxicity in various model systems. Several explanations are possible:

1. DPR inclusions are not actually involved in the *C9orf72* pathomechanism but only TDP-43 inclusions. The strongest counterarguments are rare *C9orf72* cases without TDP-43 pathology and abundant DPR pathology [2, 36, 38, 42]. In addition, DPR pathology seems to precede TDP-43 pathology, although it is not spatially correlated [2, 33]. Moreover, introducing stop codons into the GGGGCC repeat expansion prevented toxicity in the fly model, strongly arguing for a critical role of DPR proteins [35]. Methylation in the *C9orf72* promoter region is associated with reduced RNA foci and DPR pathology and prolonged disease duration presumably by inhibition of repeat transcription, which supports a toxic gain of function pathomechanism [4, 28, 44].
2. Soluble DPR proteins, rather than inclusions, may cause neurodegeneration. Although diffuse poly-GA coalesces into inclusions in cell culture systems [59], it remains unclear whether DPR proteins in cells with diffuse staining patterns cause enhanced toxicity. Soluble poly-GR/PR may interfere with the overall cellular RNA metabolism [23]. Intercellular spreading of DPR proteins may trigger pathogenic mechanisms leading to TDP-43 phosphorylation or seed TDP-43 aggregation in a non-cell autonomous manner. Spreading and seeding have been reported for other intracellular aggregating proteins in neurodegenerative diseases, but have not been claimed to be the main source of toxicity [22, 53].
3. Finally and most likely, a combination of DNA•RNA hybrids, RNA foci and protein toxicity, together with a potential *C9orf72* haploinsufficiency and unknown cell type-specific susceptibility factors are responsible for the selective neurodegeneration in certain brain regions in *C9orf72* mutation carriers. This is supported by a very recent mouse model showing TDP-43 pathology, neurodegeneration, RNA foci and DPR proteins upon high-level viral expression of the GGGGCC repeat [5].

This interaction of DNA/RNA toxicity and DPR toxicity may be represented by the newly described para-nucleolar DPR aggregates. Thus, models expressing both repeat RNA and DPR proteins and constant comparison with pathological analysis of patient samples are needed to elucidate the cause of neurodegeneration in *C9orf72* repeat expansion carriers, and how this can lead to either FTLD or MND.

### Electronic supplementary material

Supplementary material 1 (PDF 6712 kb)

## Appendix

Clinical contributions came from members of the German Consortium for Frontotemporal Lobar Degeneration: Adrian Danek, Janine Diehl-Schmid, Klaus Fassbender, Hans Förstl, Johannes Kornhuber, Markus Otto.

Clinical contributions came from members of the Bavarian Brain Banking Alliance: Andres Ceballos-Baumann, Marianne Dieterich, Regina Feuerecker, Armin Giese, Hans Klünemann, Alexander Kurz, Johannes Levin, Stefan Lorenzl, Thomas Meyer, Georg Nübling, Sigrun Roeber.
