# Supplementary material for: Distribution of dipeptide repeat proteins in cellular models and C9orf72 mutation cases suggests link to transcriptional silencing
Source: Acta Neuropathol. 2015 Jun 18;130(4):537–55. doi: 10.1007/s00401-015-1450-z (PMC4575390; doi:10.1007/s00401-015-1450-z)
Supplement: Supplementary file 1 — Supplementary material 1 (PDF 6712 kb) [file 401_2015_1450_MOESM1_ESM.pdf]

Online Supplemental Materials

Distribution of dipeptide repeat proteins in cellular models and *C9orf72* mutation cases suggests link to transcriptional silencing

Martin H. Schludi, Stephanie May, Friedrich A. Grässer, Kristin Rentzsch, Elisabeth Kremmer, Clemens Küpper, Thomas Klopstock, German Consortium for Frontotemporal Lobar Degeneration, Bavarian Brain Banking Alliance, Thomas Arzberger, Dieter Edbauer

Includes:

Supplemental Figures S1-S8

Supplemental Tables S1-S2

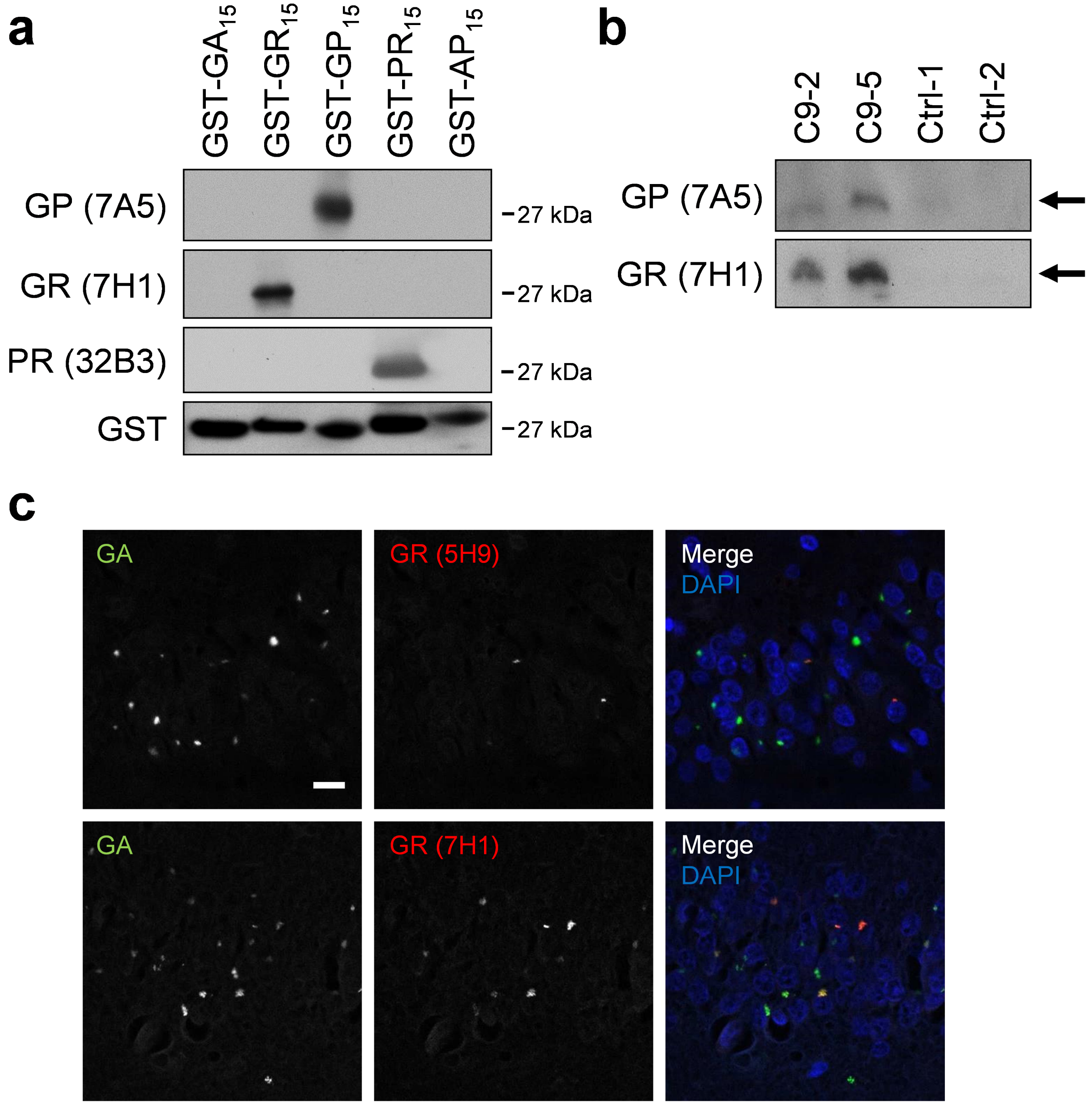

Figure S1: Monoclonal poly-GR and poly-GP antibodies are specific

(**a**) Immunoblot of 200 ng recombinant GST-GA_{15}, GST-GR_{15}, GST-GP_{15}, GST-PR_{15} and GST-AP_{15} fusion proteins with indicated antibodies as described previously (Mori et al, 2013a). (**b**) Immunoblot of the insoluble fraction of cerebellum shows poly-GP and poly-GR aggregates at the top of the gel (arrows) in *C9orf72* cases (C9) but not in healthy controls (Ctrl). Frontal cortex samples were prepared by boiling the Triton X-100 (1 %) and SDS (2 %) insoluble fraction in 4x Laemmli buffer (containing 8 % SDS) as described previously (Mori et al, 2013c). poly-PR inclusions are very rare and can only be detected by immunostaining. (**c**) Double immunofluorescence showing coaggregation of poly-GA and poly-GR in the dentate gyrus of hippocampus. Poly-GA is used as a reference for the total number of DPR inclusions. poly-GR antibody 7H1 shows more inclusions pathology than antibody 5H9. Scale bar represents 20 µm.

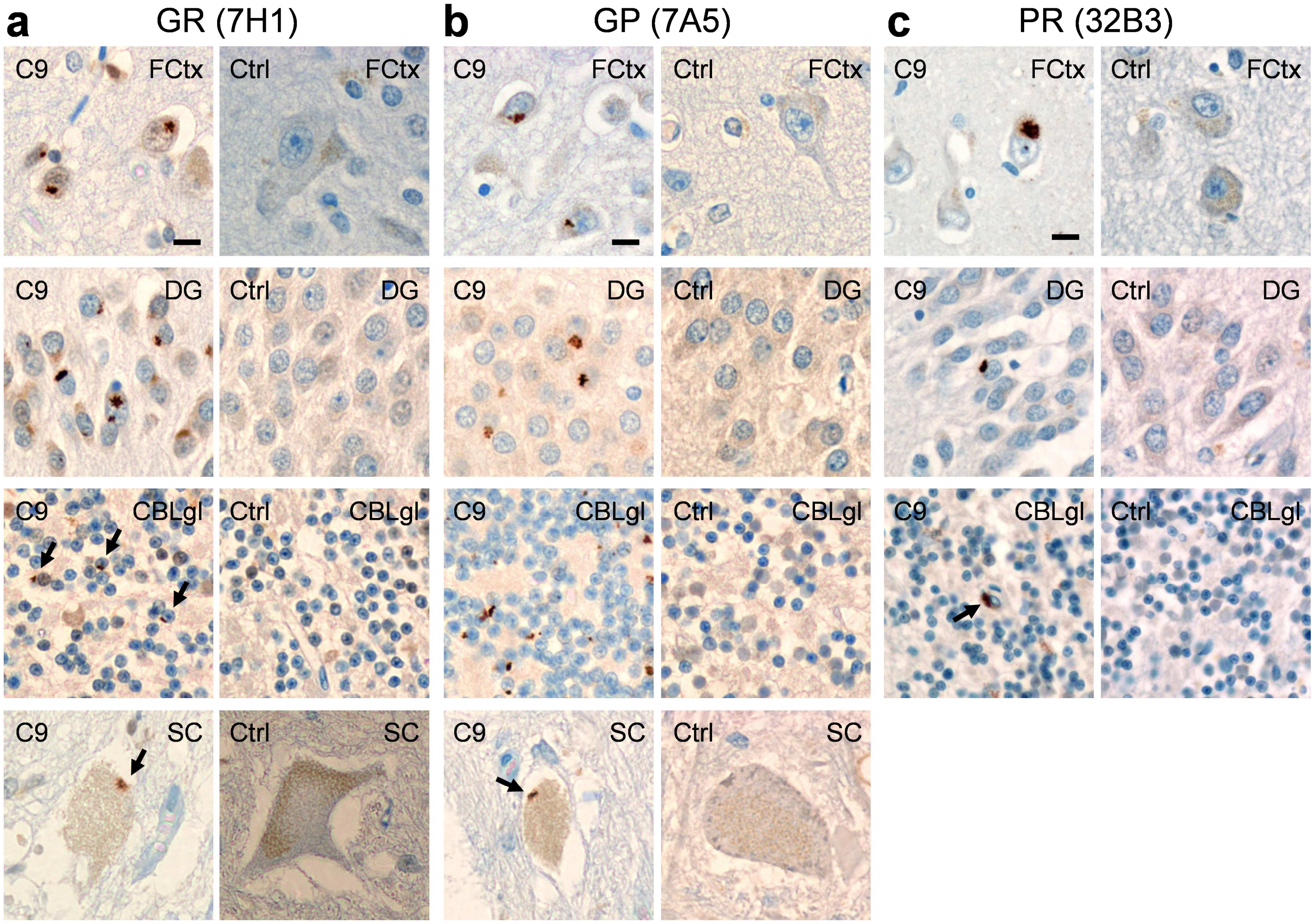

Figure S2: Poly-GR, poly-GP and poly-PR aggregates are specific for patients with *C9orf72* mutations.

Immunohistochemistry with novel monoclonal antibodies for (**a**) poly-GR (rat clone 7H1) and (**b**) poly-GP (rat clone 7A5), shows abundant inclusions in various brain regions of patients with *C9orf72* mutations (C9). Many aggregates are seen in the frontal cortex (FCtx), dentate gyrus of hippocampus (DG) and granular cell layer of cerebellum (CBLgl). Rarely, motoneurons in the spinal cord (SC) contain small intracytoplasmic poly-GR and poly-GP aggregates which are mainly localized at the edge of the cells. No inclusions are seen in a control brain (Ctrl). (**c**) Mouse poly-PR antibody 32B3 detects similar *C9orf72*-specific inclusions in FCtx, DG and CBLgl, but with much lower abundance. Poly-PR inclusions in motoneurons of the spinal cord were not detectable. Scale bar represents 20 µm.

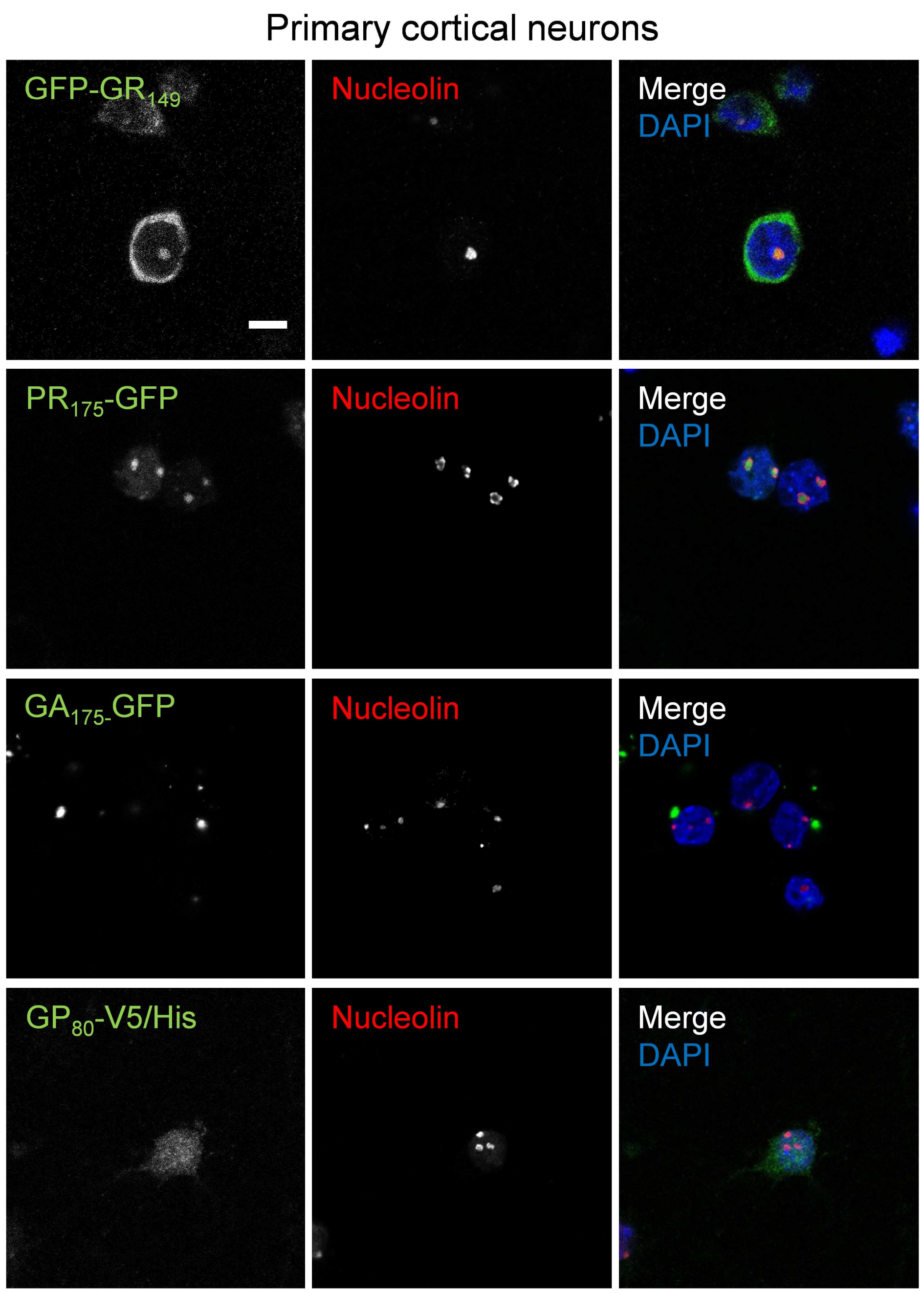

Figure S3: Expression pattern of DPR proteins in primary cortical neurons

Primary cortical neurons transduced with lentivirus expressing either GFP-GR_{149}, PR_{175}-GFP, GA_{175}-GFP or GP_{80}-V5/His (DIV6+7). Double immunofluorescence for different DPR proteins and nucleolin. Nuclei are labeled with DAPI. Single confocal sections containing the nucleolus are shown. The expression pattern of all DPR species strongly resembles the pattern seen in hippocampal neurons (Fig. 1a). Neurons expressing PR_{175}-GFP often have fragmented nucleoli. Scale bar represent 10 µm.

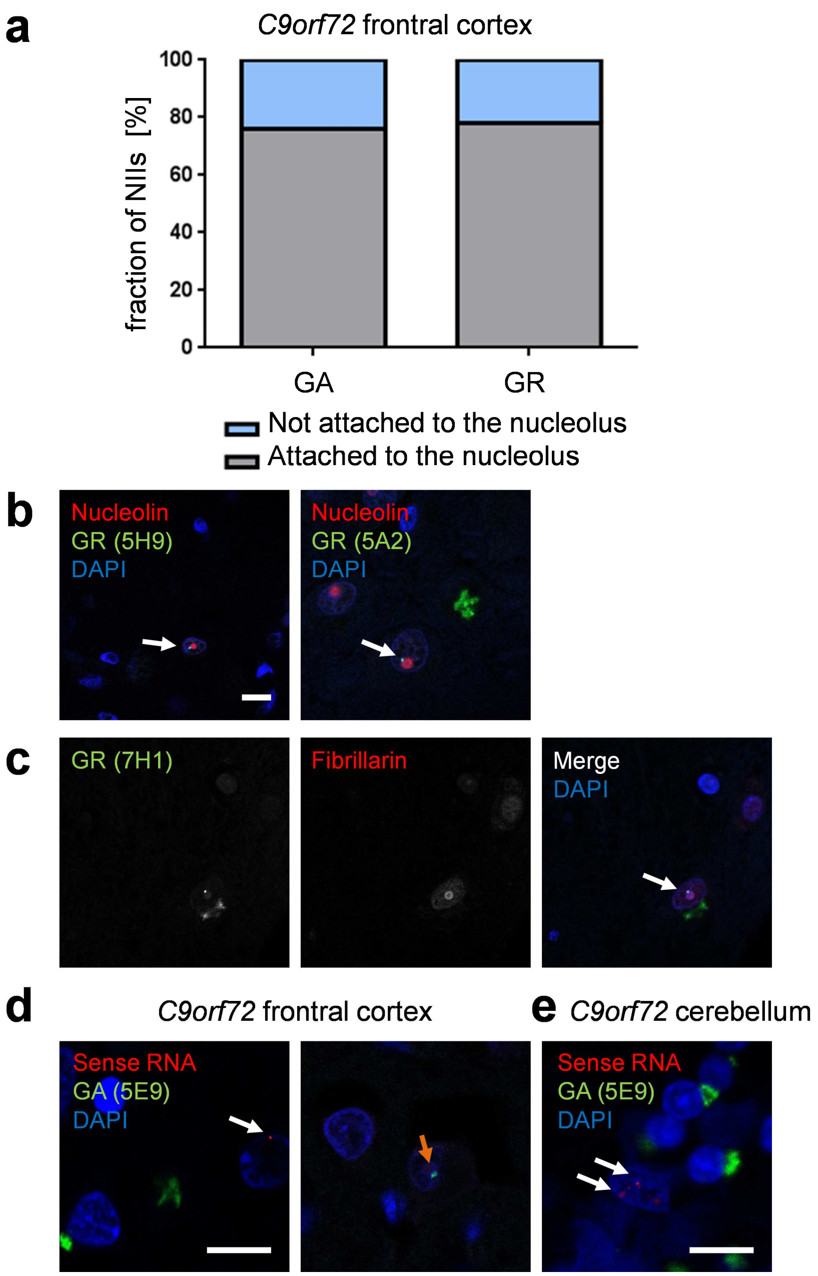

Figure S4: Poly-GR antibodies detect para-nucleolar aggregates

(**a**) Quantitative analysis of the localization of poly-GA and poly-GR NIIs was performed on double immunofluorescence stains with nucleolin in cortical areas of two *C9orf72* patients and two controls. 76% of poly-GA NIIs and 78% of poly-GR NIIs are attached to the nucleolus (50 NIIs each were analyzed). (**b**) Para-nucleolar poly-GR aggregates (arrows) are detected by two additional monoclonal poly-GR antibodies (5H9 and 5A2) as shown by double immunofluorescence with nucleolin. (**c**) Para-nucleolar poly-GR aggregates (arrow) are detected using fibrillarin as an alternative marker for nucleoli. Scale bars represent 20 µm. (**d**, **e**) Combined GGGGCC-specific *in situ* hybridization and poly-GA immunofluorescence shows no colocalization of RNA foci (white arrows) and para-nucleolar DPR inclusions (orange arrow).

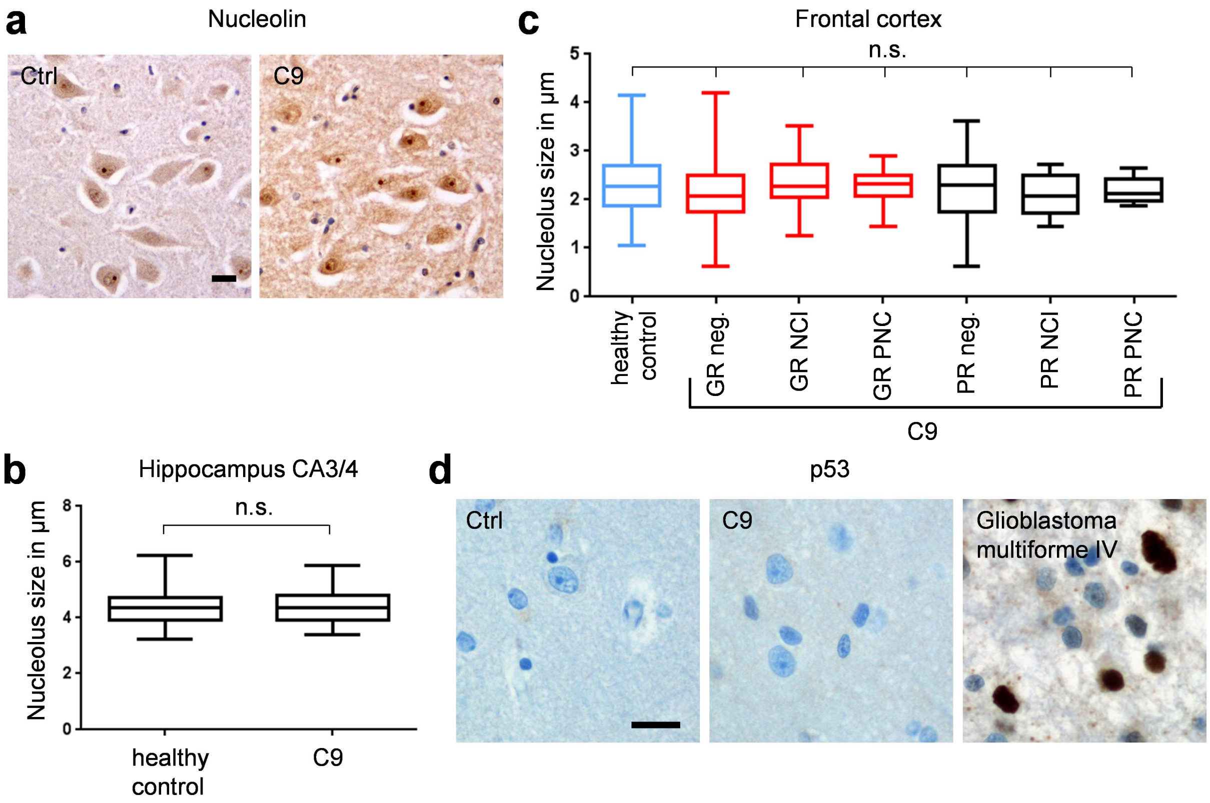

Figure S5: Analysis of nucleolar stress in *C9orf72* cases

Analysis of the hallmarks of nucleolar stress in *C9orf72* mutation patients (C9) and controls (Ctrl). (**a**) Immunohistochemistry with a nucleolin antibody reveals no difference in the shape of the nucleoli in neurons of the CA3/4 region between cases with *C9orf72* mutation and control cases. (**b**) Quantitative analysis of the average nucleolus size in the CA3/4 region performed on nucleolin immunofluorescence images reveals no difference between *C9orf72* patients and controls as well (n_{ctrl}=45, n_{C9}=55 nucleoli from two control cases and four *C9orf72* patients). The detailed analysis is described in the methods section. (**c**) Quantitative analysis of nucleolus size performed on nucleolin and poly-GR or poly-PR double immunofluorescence images in the frontal cortex. In *C9orf72* patients nucleolus size was analyzed separately for cells without DPR inclusions, with neuronal cytoplasmic inclusions (NCI) or inclusions in a para-nucleolar compartment (PNC) using MetaMorph software. n_{ctrl}=91, $n_{GR\ NCI}$=40, $n_{GR\ PNC}$=12, $n_{GR\ neg.}$=126, $n_{PR\ NCI.}$=12, $n_{PR\ PNC}$=5, $n_{PR\ neg.}$=78 nucleoli were investigated from two control cases and two *C9orf72* patients. Box plot shows mean, first and third quartile. Whiskers represent minimum and maximum. (**d**) Immunohistochemistry of frontal cortex using p53 antibodies in *C9orf72* patients, a healthy control and a case with glioblastoma as a positive control. Stress-indicative nucleolar accumulation of p53 in the nucleolus is not detected in *C9orf72* cases. Scale bars represent 20 µm.

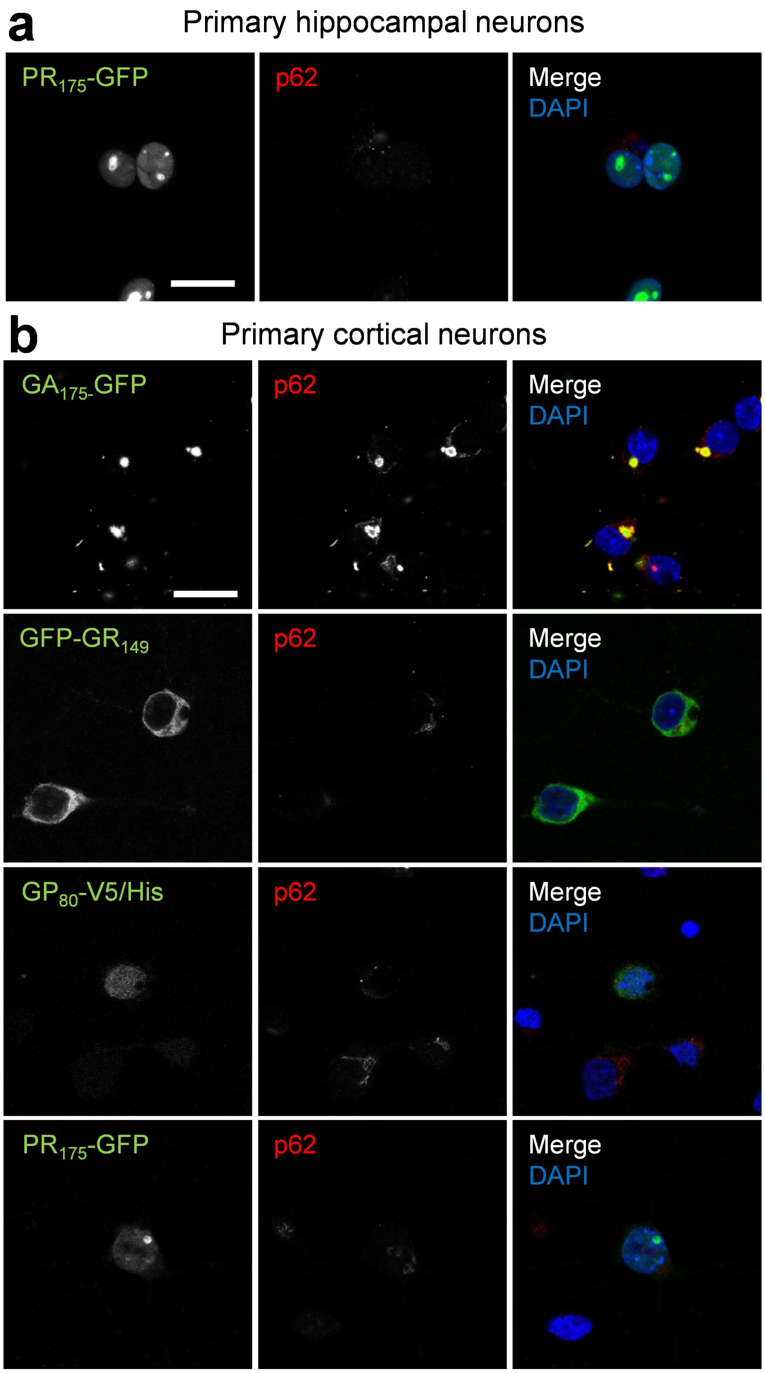

Figure S6: Colocalization of DPR proteins with p62 in primary neurons

Double immunofluorescence for DPR proteins and p62. Nuclei are labeled with DAPI. (**a**) PR_{175}-GFP expressed in primary hippocampal neurons does not colocalize with p62 (DIV6+7). (**b**) Primary cortical neurons transduced with GA_{175}-GFP, GFP-GR_{149}, GP_{80}-V5/His or PR_{175}-GFP (DIV6+7). p62 co-aggregates only with cytoplasmic and intranuclear poly-GA inclusions, but not with poly-GR, poly-GP and poly-GR. Scale bars represent 20 µm.

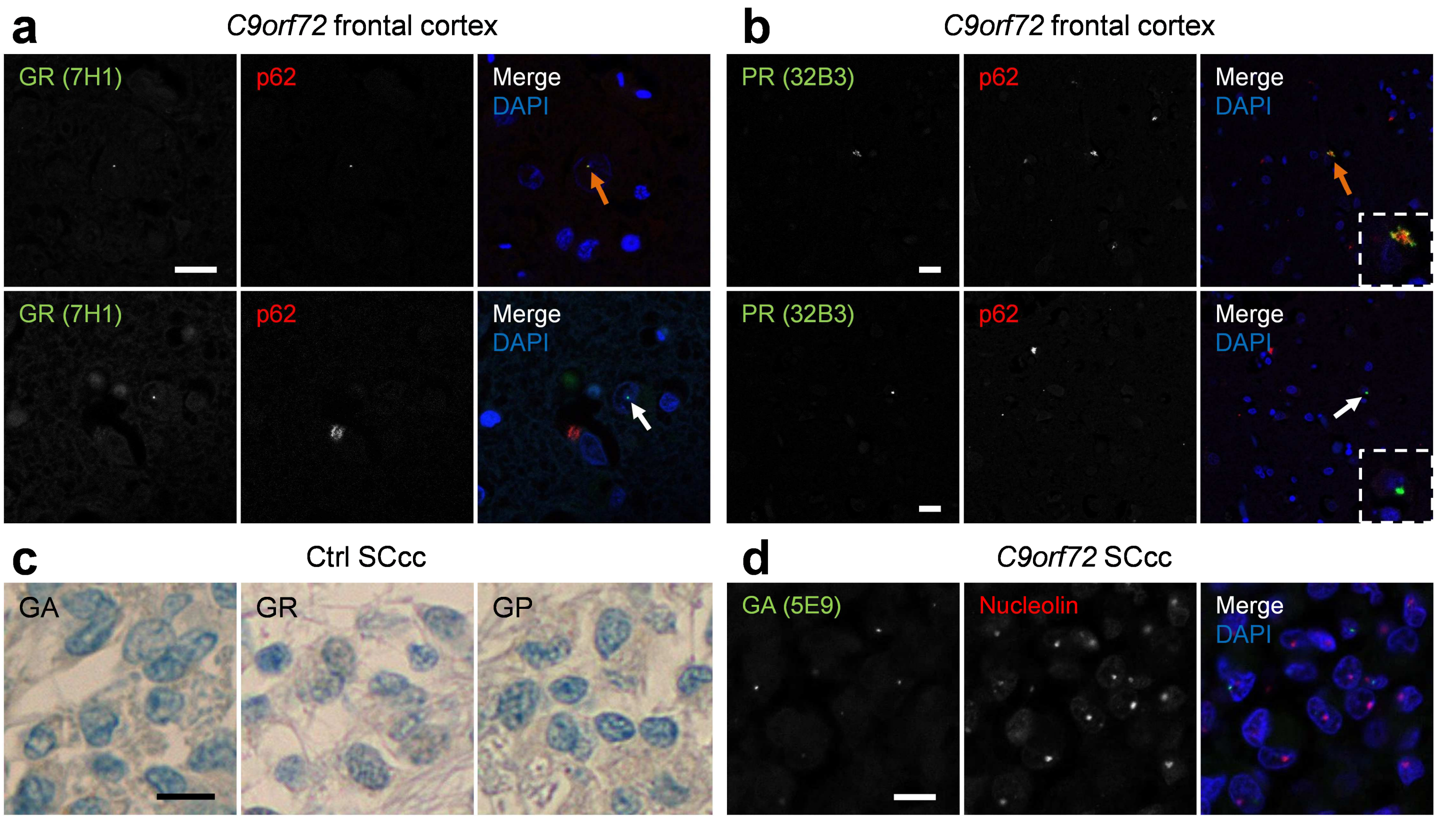

Figure S7: p62 negative DPR inclusions are rare. DPR aggregates in the central canal of the spinal cord are not found in control cases and do not localize close to the nucleolus in *C9orf72* patients

(**a**) Double immunofluorescence with poly-GR (7H1) and p62 in frontal cortex. Aside from p62 positive intranuclear inclusions (orange arrow), p62 negative intranuclear poly-GR inclusions also exist (white arrow), but are very rare. (**b**) In frontal cortex of *C9orf72* mutation patients most poly-PR inclusions are positive for p62 (orange arrow). Rarely a p62 negative poly-PR inclusion for can be found (white arrows). (**c**) Immunohistochemistry with antibodies against poly-GA (5E9), poly-GR (7H1) and poly-GP (7A5) shows no DPR inclusions in the central canal of the spinal cord (SCcc) in an FTLD-MND-FUS case (FUS-1). (**d**) Double immunofluorescence with poly-GA and nucleolin shows that DPR inclusions are randomly distributed within the nuclei in SCcc glial cells. Scale bars represent 20 µm.

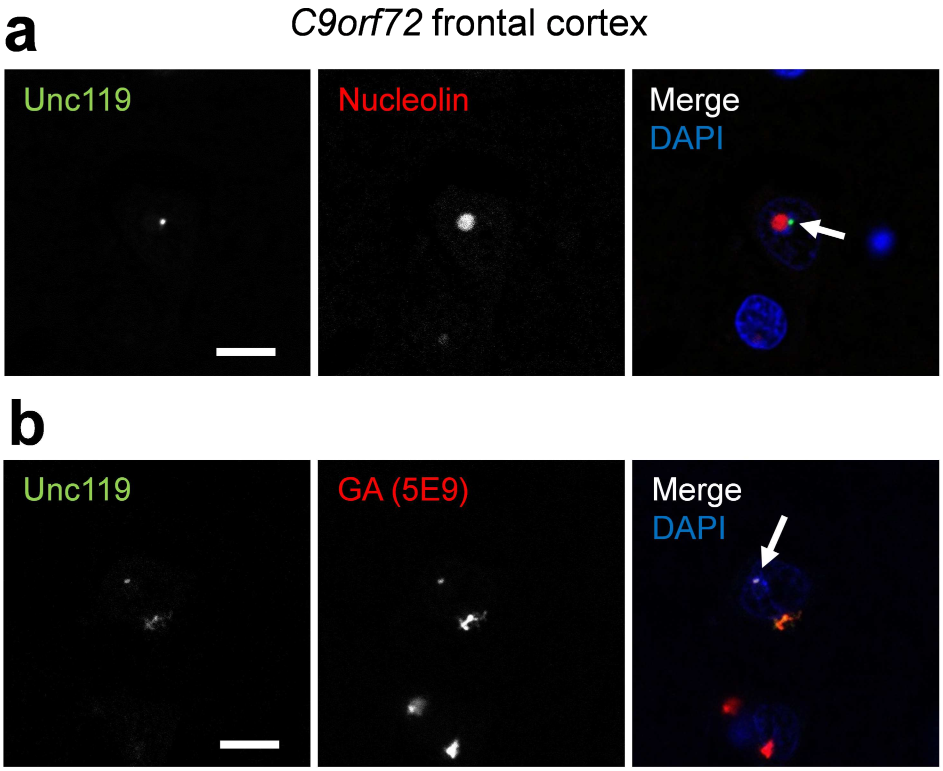

Figure S8: Unc119 is colocalized with poly-GA in para-nucleolar inclusions

Double immunofluorescence with Unc119 and nucleolin, poly-GA or p62 in frontal cortex. (**a**) Some Unc119 inclusions show para-nucleolar localization (arrow). (**b**) Intranuclear Unc119 is colocalized with poly-GA (arrow).

Table S1: Semiquantitative assessment of DPR and Unc119 pathology in five *C9orf72* cases

Raw data for Fig. 5 and 8a. The numbers represent the categories „none" (0), "few" (1), "some" (2), "many" (3) and "abundant" (4). The analysis is described in detail in the method section. Abbreviations as in Fig. 5.

GA immunoreactive inclusions

Case no.	FCtx			MCtx			CCtx			PCtx			TCtx			Octx			DG			CA3/4			SubCA1/2		
	NCI	NII	DN	NCI	NII	DN	NCI	NII	DN	NCI	NII	DN	NCI	NII	DN	NCI	NII	DN	NCI	NII	DN	NCI	NII	DN	NCI	NII	DN
C9-1	4	2	2	3	2	1	3	2	1	4	2	1	4	2	1	4	2	1	3	2	1	3	1	2	2	1	0
C9-2	4	2	1	4	2	2	4	2	2	4	2	2	4	2	1	4	2	2	4	2	1	4	2	3	3	1	2
C9-3	3	2	1	3	1	2	3	2	2	3	1	1	3	2	2	3	2	2	2	2	1	2	1	2	2	1	1
C9-4	3	1	0	3	2	2	3	2	1	4	2	2	4	2	2	4	2	1	3	1	1	3	1	2	2	1	1
C9-5	4	2	1	4	2	2	4	2	2	4	2	1	4	2	2	4	2	2	3	2	1	3	2	2	3	1	2
Mean	3,6	1,8	1,0	3,4	1,8	1,8	3,4	2,0	1,6	3,8	1,8	1,4	3,8	2,0	1,6	3,8	2,0	1,6	3,0	1,8	1,0	3,0	1,4	2,2	2,4	1,0	1,2

	CBLgl			CBLml			CBLpcl			Cd			Pu			Acc			Pa			BC			priEnt		
	NCI	NII	DN	NCI	NII	DN	NCI	NII	DN	NCI	NII	DN	NCI	NII	DN	NCI	NII	DN	NCI	NII	DN	NCI	NII	DN	NCI	NII	DN
C9-1	3	2	3	2	2	4	2	0	0	2	1	0	2	1	0	2	1	0	2	1	0	1	0	0	4	2	1
C9-2	4	4	4	4	2	4	3	1	0	3	1	1	2	1	0	3	1	0	2	1	0	1	0	0	4	2	2
C9-3	2	1	3	3	1	4	2	1	1	1	0	0	1	1	0	2	1	0	0	0	0	0	0	0	4	2	2
C9-4	2	1	3	2	1	4	2	0	0	1	0	1	1	1	0	1	1	1	1	1	0	1	0	0	4	2	2
C9-5	3	2	2	3	1	3	2	0	0	2	1	1	2	1	1	3	1	1	1	0	1	1	0	0	4	2	2
Mean	2,8	2,0	3,0	2,8	1,4	3,8	2,2	0,4	0,2	1,8	0,6	0,6	1,6	1,0	0,2	2,2	1,0	0,4	1,2	0,6	0,2	0,8	0,0	0,0	4,0	2,0	1,8

	preEnt			Am			Th			SNc			STN			LC			DRN			PN			N XII		
	NCI	NII	DN	NCI	NII	DN	NCI	NII	DN	NCI	NII	DN	NCI	NII	DN	NCI	NII	DN	NCI	NII	DN	NCI	NII	DN	NCI	NII	DN
C9-1	3	1	1	3	2	1										0	0	0	2	0	0	1	0	0	0	0	0
C9-2	4	1	2	4	2	2	4	2	0	1	1	0	1	0	0	0	0	0	2	1	0	1	0	0	1	0	0
C9-3	3	2	2	3	1	1	2	1	0	1	0	0	1	0	0	0	0	0	1	0	0	1	0	0			
C9-4	3	2	1	3	1	0	3	1	0	1	0	0	1	0	0	0	0	0	2	0	0	0	0	0			
C9-5	3	2	2	3	1	1	3	2	1	2	1	0	2	0	0	0	0	0	2	1	0	0	0	0	1	1	0
Mean	3,2	1,6	1,6	3,2	1,4	1,0	3,0	1,5	0,3	1,3	0,5	0,0	1,3	0,0	0,0	0,0	0,0	0,0	1,8	0,4	0,0	0,6	0,0	0,0	0,7	0,3	0,0

	IO			SCAc			SCPc			SCAt			SCPt			SCAl			SCPl			SCAs			SCPs		
	NCI	NII	DN	NCI	NII	DN	NCI	NII	DN	NCI	NII	DN	NCI	NII	DN	NCI	NII	DN	NCI	NII	DN	NCI	NII	DN	NCI	NII	DN
C9-1	0	0	0	0	0	0	1	0	0	0	0	0	1	0	0	2	0	0	2	0	0	1	0	0	1	0	0
C9-2	1	0	0	1	2	1	2	2	0	2	2	0	2	2	0	1	2	1	2	2	0	1	2	0	1	2	0
C9-3				1	0	0	1	0	0																		
C9-4				1	0	0	0	0	0	0	0	0	1	0	0	0	0	0	1	0	0	0	0	0	1	0	0
C9-5	0	0	0	1	0	0	1	1	0	0	0	0	1	0	0	1	1	0	2	1	0	1	0	0	2	0	0
Mean	0,3	0,0	0,0	0,8	0,4	0,2	1,0	0,6	0,0	0,5	0,5	0,0	1,3	0,5	0,0	1,0	0,8	0,3	1,8	0,8	0,0	0,8	0,5	0,0	1,3	0,5	0,0

GR immunoreactive inclusions

Case no.	FCtx			MCtx			CCtx			PCtx			TCtx			Octx			DG			CA3/4			SubCA1/2		
	NCI	NII	DN	NCI	NII	DN	NCI	NII	DN	NCI	NII	DN	NCI	NII	DN	NCI	NII	DN	NCI	NII	DN	NCI	NII	DN	NCI	NII	DN
C9-1	3	1	0	2	1	0	2	2	0	2	1	0	2	1	0	2	1	0	3	2	0	3	2	0	2	1	1
C9-2	3	2	0	2	1	0	3	1	0	3	1	0	3	1	0	3	1	0	3	2	0	4	2	0	3	1	0
C9-3	2	1	0	2	0	0	2	1	0	2	0	0	2	1	0	2	0	1	2	1	0	2	1	1	2	1	1
C9-4	2	1	0	2	1	0	2	1	0	2	1	0	2	1	0	2	1	0	2	1	0	3	1	0	2	1	0
C9-5	3	1	0	2	1	1	2	1	0	3	1	0	3	2	0	3	1	1	2	2	0	2	1	1	2	1	2
Mean	2,6	1,2	0,0	2,0	0,8	0,2	2,2	1,2	0,0	2,4	0,8	0,0	2,4	1,2	0,0	2,4	0,8	0,4	2,4	1,6	0,0	2,8	1,4	0,4	2,2	1,0	0,8

	CBLgl			CBLml			CBLpcl			Cd			Pu			Acc			Pa			BC			priEnt		
	NCI	NII	DN	NCI	NII	DN	NCI	NII	DN	NCI	NII	DN	NCI	NII	DN	NCI	NII	DN	NCI	NII	DN	NCI	NII	DN	NCI	NII	DN
C9-1	2	1	0	2	1	0	2	0	0	1	0	0	1	1	0	1	0	0	0	0	0	0	0	0	3	2	0
C9-2	2	1	0	2	1	1	2	0	0	1	0	0	1	0	0	1	0	0	1	0	0	0	0	0	3	1	0
C9-3	1	0	0	2	1	1	2	0	0	1	0	0	1	0	0	1	0	0	0	0	0	0	0	0	2	1	0
C9-4	1	0	0	2	1	0	2	0	0	0	0	0	1	1	0	1	0	1	0	0	0	0	0	0	2	1	0
C9-5	2	1	1	2	2	0	2	0	0	0	0	0	1	0	0	1	0	0	1	1	0	0	0	0	3	1	0
Mean	1,6	0,6	0,2	2,0	1,2	0,4	2,0	0,0	0,0	0,6	0,0	0,0	1,0	0,4	0,0	1,0	0,0	0,2	0,4	0,2	0,0	0,0	0,0	0,0	2,6	1,2	0,0

	preEnt			Am			Th			SNc			STN			LC			DRN			PN			N XII		
	NCI	NII	DN	NCI	NII	DN	NCI	NII	DN	NCI	NII	DN	NCI	NII	DN	NCI	NII	DN	NCI	NII	DN	NCI	NII	DN	NCI	NII	DN
C9-1	2	1	0	2	1	0										0	0	0	0	0	0	0	0	0	0	0	0
C9-2	2	1	0	3	2	1	3	2	0	1	0	0	0	0	0	0	0	0	1	0	0	0	0	0	1	0	0
C9-3	1	1	0	2	1	0	1	1	0	0	0	0	0	0	0	0	0	0	1	0	0	1	1	0			
C9-4	1	0	0	2	1	0	1	1	0	0	0	0	0	0	0	0	0	0	0	0	0	0	0	0			
C9-5	2	1	0	3	1	0	2	1	0	1	0	0	0	0	0	0	0	0	1	0	0	1	0	0	1	0	0
Mean	1,6	0,8	0,0	2,4	1,2	0,2	1,8	1,3	0,0	0,5	0,0	0,0	0,0	0,0	0,0	0,0	0,0	0,0	0,6	0,0	0,0	0,4	0,2	0,0	0,7	0,0	0,0

	IO			SCAc			SCPc			SCAt			SCPt			SCAl			SCPl			SCAs			SCPs		
	NCI	NII	DN	NCI	NII	DN	NCI	NII	DN	NCI	NII	DN	NCI	NII	DN	NCI	NII	DN	NCI	NII	DN	NCI	NII	DN	NCI	NII	DN
C9-1	1	0	0	0	0	0	0	0	0	0	0	0	1	0	0	1	0	0	0	0	0	0	0	0	1	0	0
C9-2	0	0	0	1	0	0	0	0	0	0	0	0	0	0	0	0	0	0	1	0	0	0	0	0	0	0	0
C9-3				0	0	0	1	0	0																		
C9-4				1	0	0	1	0	0	0	0	0	1	0	1	1	0	0	1	0	0	0	0	0	0	0	0
C9-5	1	0	0	0	0	0	0	0	0	0	0	0	1	0	0	1	0	1	0	0	0	1	0	0	1	0	0
Mean	0,7	0,0	0,0	0,4	0,0	0,0	0,4	0,0	0,0	0,0	0,0	0,0	0,8	0,0	0,3	0,8	0,0	0,3	0,5	0,0	0,0	0,3	0,0	0,0	0,5	0,0	0,0

GP immunoreactive inclusions

Case no.	FCtx			MCtx			CCtx			PCtx			TCtx			Octx			DG			CA3/4			SubCA1/2		
	NCI	NII	DN	NCI	NII	DN	NCI	NII	DN	NCI	NII	DN	NCI	NII	DN	NCI	NII	DN	NCI	NII	DN	NCI	NII	DN	NCI	NII	DN
C9-1	3	1	0	3	1	1	2	1	0	2	1	1	2	1	0	3	1	1	3	2	1	3	1	1	2	1	1
C9-2	3	1	1	2	1	1	3	2	0	2	1	0	3	2	0	3	2	1	3	2	1	4	2	1	2	1	2
C9-3	2	0	1	2	1	0	2	1	0	2	0	0	3	0	0	2	1	1	2	1	0	2	2	0	2	1	0
C9-4	3	1	1	3	1	1	2	1	0	3	1	0	2	0	0	3	1	1	2	1	0	2	1	0	2	1	1
C9-5	3	1	1	2	1	0	3	2	1	3	1	1	3	1	1	2	1	0	3	2	1	3	2	1	2	1	2
Mean	2,8	0,8	0,8	2,4	1,0	0,6	2,4	1,4	0,2	2,4	0,8	0,4	2,6	0,8	0,2	2,6	1,2	0,8	2,6	1,6	0,6	2,8	1,6	0,6	2,0	1,0	1,2

	CBLgl			CBLml			CBLpcl			Cd			Pu			Acc			Pa			BC			priEnt		
	NCI	NII	DN	NCI	NII	DN	NCI	NII	DN	NCI	NII	DN	NCI	NII	DN	NCI	NII	DN	NCI	NII	DN	NCI	NII	DN	NCI	NII	DN
C9-1	2	2	1	2	1	3	2	0	0	1	0	0	1	1	0	1	1	0	1	0	1	0	0	0	3	1	1
C9-2	3	2	2	4	1	3	2	0	0	1	0	0	1	1	0	1	0	0	1	0	0	1	0	0	3	2	0
C9-3	2	1	2	2	1	2	2	0	0	1	0	0	1	0	0	1	0	0	1	0	0	1	0	0	3	1	1
C9-4	1	0	0	2	1	2	2	0	0	0	0	0	1	0	0	0	0	1	1	1	0	1	0	0	2	1	0
C9-5	2	2	2	2	2	3	2	0	0	0	0	0	1	0	0	0	0	0	1	0	0	0	0	0	3	1	1
Mean	2,0	1,4	1,4	2,4	1,2	2,6	2,0	0,0	0,0	0,6	0,0	0,0	1,0	0,4	0,0	0,6	0,2	0,2	1,0	0,2	0,2	0,6	0,0	0,0	2,8	1,2	0,6

	preEnt			Am			Th			SNc			STN			LC			DRN			PN			N XII		
	NCI	NII	DN	NCI	NII	DN	NCI	NII	DN	NCI	NII	DN	NCI	NII	DN	NCI	NII	DN	NCI	NII	DN	NCI	NII	DN	NCI	NII	DN
C9-1	2	1	0	2	1	1										0	0	0	2	0	1	0	0	0	0	0	0
C9-2	2	1	0	3	1	0	3	2	0	2	1	0	0	0	0	0	0	0	1	0	0	0	0	0	1	0	0
C9-3	2	1	0	2	1	0	2	2	1	0	0	0	1	0	0	0	0	0	1	1	0	0	0	0			
C9-4	1	0	0	2	1	0	2	1	0	0	0	0	1	0	0	0	1	0	2	0	0	0	0	0			
C9-5	2	1	0	3	1	0	2	1	0	0	0	0	0	0	0	1	0	0	1	1	0	1	0	0	0	0	0
Mean	1,8	0,8	0,0	2,4	1,0	0,2	2,3	1,5	0,3	0,5	0,3	0,0	0,5	0,0	0,0	0,2	0,2	0,0	1,4	0,4	0,2	0,2	0,0	0,0	0,3	0,0	0,0

	IO			SCAc			SCPc			SCAt			SCPt			SCAl			SCPl			SCAs			SCPs		
	NCI	NII	DN	NCI	NII	DN	NCI	NII	DN	NCI	NII	DN	NCI	NII	DN	NCI	NII	DN	NCI	NII	DN	NCI	NII	DN	NCI	NII	DN
C9-1	0	0	0	0	0	1	1	1	0	0	0	0	1	0	1	1	0	1	1	0	2	0	0	0	0	0	0
C9-2	1	0	0	1	1	0	1	1	0	0	0	0	1	1	1	1	1	0	1	1	0	0	0	0	0	0	0
C9-3				1	0	0	1	0	0																		
C9-4				0	0	0	0	0	0	0	0	0	0	0	0	1	0	0	1	0	0	0	0	0	0	0	0
C9-5	0	0	0	1	0	0	1	1	0	0	0	0	1	0	0	1	0	0	1	0	0	1	0	0	1	0	1
Mean	0,3	0,0	0,0	0,6	0,2	0,2	0,8	0,6	0,0	0,0	0,0	0,0	0,8	0,3	0,5	1,0	0,3	0,3	1,0	0,3	0,5	0,3	0,0	0,0	0,3	0,0	0,3

Unc119 immunoreactive inclusions

Case no.	FCtx			MCtx			CCtx			PCtx			TCtx			Octx			DG			CA3/4			SubCA1/2		
	NCI	NII	DN	NCI	NII	DN	NCI	NII	DN	NCI	NII	DN	NCI	NII	DN	NCI	NII	DN	NCI	NII	DN	NCI	NII	DN	NCI	NII	DN
C9-1	3	2	1	2	1	0	2	1	0	2	1	1	2	1	1	2	1	1	2	1	0	2	1	0	2	1	0
C9-2	3	1	1	3	1	0	3	1	0	3	1	1	2	1	0	2	1	0	3	2	0	3	1	1	2	1	1
C9-3	2	1	0	2	1	1	2	1	1	2	1	1	2	1	1	2	0	1	2	1	0	2	1	1	2	1	0
C9-4	2	1	0	2	0	0	2	1	0	2	0	1	2	0	0	2	1	1	2	1	0	3	0	0	2	0	0
C9-5	3	1	1	3	1	1	3	1	1	3	0	1	3	1	1	3	1	0	2	2	0	2	0	2	2	0	2
Mean	2,6	1,2	0,6	2,4	0,8	0,4	2,4	1,0	0,4	2,4	0,6	1,0	2,2	0,8	0,6	2,2	0,8	0,6	2,2	1,4	0,0	2,4	0,6	0,8	2,0	0,6	0,6

	CBLgl			CBLml			CBLpcl			Cd			Pu			Acc			Pa			BC			priEnt		
	NCI	NII	DN	NCI	NII	DN	NCI	NII	DN	NCI	NII	DN	NCI	NII	DN	NCI	NII	DN	NCI	NII	DN	NCI	NII	DN	NCI	NII	DN
C9-1	2	0	1	2	1	2	2	0	0	1	0	0	1	0	0	1	0	0	1	0	0	0	0	0	3	1	1
C9-2	2	1	2	3	1	2	3	0	0	2	1	0	2	1	0	2	1	0	1	1	0	1	0	1	3	1	0
C9-3	2	1	1	2	1	1	2	0	0	1	0	0	1	0	0	1	0	0	1	0	0	0	0	0	3	1	1
C9-4	1	0	0	2	1	0	2	0	0	0	0	0	1	0	0	1	0	0	1	0	0	9	0	0	2	1	0
C9-5	2	1	1	3	1	2	2	0	0	1	0	0	2	0	0	2	1	1	0	0	1	0	0	0	3	1	1
Mean	1,8	0,6	1,0	2,4	1,0	1,4	2,2	0,0	0,0	1,0	0,2	0,0	1,4	0,2	0,0	1,4	0,4	0,2	0,8	0,2	0,2	2,0	0,0	0,2	2,8	1,0	0,6

	preEnt			Am			Th			SNc			STN			LC			DRN			PN			N XII		
	NCI	NII	DN	NCI	NII	DN	NCI	NII	DN	NCI	NII	DN	NCI	NII	DN	NCI	NII	DN	NCI	NII	DN	NCI	NII	DN	NCI	NII	DN
C9-1	2	0	0	2	1	0										0	0	0	1	0	0	0	0	0	0	0	0
C9-2	2	1	0	3	1	1	3	1	0	1	0	0	1	0	0	0	0	0	2	0	0	1	0	0	0	0	0
C9-3	2	1	1	2	1	0	2	1	0	1	0	0	0	0	0	0	0	0	1	0	0	0	0	0			
C9-4	1	0	0	2	0	0	2	0	0	0	0	0	1	0	0	0	0	0	1	0	0	0	0	0			
C9-5	2	1	0	3	1	1	2	0	0	1	0	0	1	0	0	0	0	0	1	0	0	1	0	0	0	0	0
Mean	1,8	0,6	0,2	2,4	0,8	0,4	2,3	0,5	0,0	0,8	0,0	0,0	0,8	0,0	0,0	0,0	0,0	0,0	1,2	0,0	0,0	0,4	0,0	0,0	0,0	0,0	0,0

	IO			SCAc			SCPc			SCAt			SCPt			SCAl			SCPl			SCAs			SCPs		
	NCI	NII	DN	NCI	NII	DN	NCI	NII	DN	NCI	NII	DN	NCI	NII	DN	NCI	NII	DN	NCI	NII	DN	NCI	NII	DN	NCI	NII	DN
C9-1	0	0	0	1	0	1	1	0	0	0	0	0	0	1	1	1	0	1	0	0	0	0	0	0	1	0	0
C9-2	1	0	0	1	1	1	0	0	0	0	0	0	1	0	0	0	0	0	1	0	0	1	0	0	0	0	0
C9-3				0	0	0	1	0	0																		
C9-4				0	0	0	0	0	0	0	0	0	1	1	0	0	0	0	0	1	0	0	0	0	0	0	0
C9-5	0	0	0	0	0	0	1	0	1	1	0	0	1	0	0	1	0	0	1	0	0	1	0	0	1	0	0
Mean	0,3	0,0	0,0	0,4	0,2	0,4	0,6	0,0	0,2	0,3	0,0	0,0	0,8	0,5	0,3	0,5	0,0	0,3	0,5	0,3	0,0	0,5	0,0	0,0	0,5	0,0	0,0

Table S2: Quantitative assessment of DPR and Unc119 pathology in 14 *C9orf72* cases

Raw data for the quantitative analysis of inclusion pathology in 14 *C9orf72* mutation cases shown in Fig. 6, 8a and 8b. Three to twelve pictures were taken from representative areas of each region. In cortical areas pictures were taken in a columnar orientation covering layer I to VI. The total number of inclusions was divided by the number of pictures taken. For details see methods section, for abbreviations Fig. 6.

GR (NCI + NII)

Case no.	FCtx	MCtx	OCtx	DG	CA3/4	CBLgl	CBLml
C9-1	11.5	5.1	6.0	48.5	10.0	29.5	6.0
C9-2	17.4	5.0	22.8	102.0	16.7	20.0	9.7
C9-3	3.4	1.5	3.5	6.7	14.7	8.0	5.7
C9-4	4.0	6.0	6.6	14.0	12.0	0.7	0.7
C9-5	15.2	5.0	11.0	31.0	13.0	37.5	13.0
C9-6	7.1		0.5			1.7	4.7
C9-7	4.1		18.3	64.7	23.7	3.7	2.3
C9-8	2.6		2.0	2.7	3.3	1.0	3.0
C9-9	5.0		6.8	34.7	10.0	0.3	1.0
C9-10	2.4		0.0	18.7	27.7	0.0	3.0
C9-11	2.5		0.3	2.0	1.0	1.3	0.7
C9-12	7.0		1.6	13.0	7.0	1.0	0.0
C9-13	22.6		32.4			34.3	12.7
C9-14	7.0	5.4	9.0	30.0	10.3	10.0	7.7

PR (NCI + NII)

Case no.	FCtx	MCtx	OCtx	DG	CA3/4	CBLgl	CBLml
C9-1	0.1	0.0	0.2	1.3	2.7	0.0	0.0
C9-2	0.1	0.0	0.0	0.7	1.0	0.3	0.0
C9-3	0.1	0.0	0.0	0.0	0.0	1.0	0.0
C9-4	0.0	0.2	0.2	0.0	0.0	0.0	0.0
C9-5	0.0	0.0	0.0	0.0	0.0	0.0	0.0
C9-6	0.0		0.0			0.0	0.0
C9-7	0.0		0.0	0.3	2.3	0.3	0.3
C9-8	0.5		0.0	0.0	0.7	0.0	0.0
C9-9	0.2		0.0	0.3	0.3	0.3	0.0
C9-10	0.0		0.0			0.3	0.0
C9-11	0.0		0.0	0.0	0.3	1.0	0.0
C9-12	0.0		0.0	0.3	0.3	0.7	0.0
C9-13	0.1		0.1			0.7	0.0
C9-14	0.0	0.0	0.0	0.0	0.0	0.0	0.0

GA (NCI + NII)

Case no.	FCtx	MCtx	OCtx	DG	CA3/4	CBLgl	CBLml
C9-1	10.6	6.5	9.8	70.7	30.7	85.3	16.0
C9-2	46.7	34.8	75.5	368.3	40.3	364.3	61.0
C9-3	14.6	14.0	16.2	26.0	13.0	83.0	17.7
C9-4	6.7	4.6	7.9	47.0	29.7	180.0	15.0
C9-5	48.6	25.4	79.1	80.3	28.0	183.7	39.0
C9-6	23.7		21.5			153.3	26.0
C9-7	20.0		15.2	94.0	21.3	338.5	24.0
C9-8	44.8		51.8	95.0	19.0	473.0	54.3
C9-9	14.0		24.0	56.3	11.5	315.0	35.0
C9-10	34.2		55.5	112.7	40.0	123.0	58.3
C9-11	30.8		3.3	47.0	12.7	283.0	31.0
C9-12	20.2		19.4	55.0	21.0	179.0	20.7
C9-13	25.2		46.2			437.3	29.0
C9-14	16.4	15.4	26.5	87.0	17.7	119.0	15.0

Unc119 (NCI + NII)

Case no.	FCtx	MCtx	OCtx	DG	CA3/4	CBLgl	CBLml
C9-1	5.9	2.5	3.2	16.0	7.0	16.0	6.0
C9-2	16.8	17.7	16.0	141.5	27.7	133.0	25.7
C9-3	6.2	4.4	5.0	13.7	8.3	20.0	3.3
C9-4	2.0	2.0	2.2	5.8	16.6	7.3	2.7
C9-5	14.2	14.8	19.6	27.3	16.0	100.3	22.3
C9-6	12.8		6.5			39.0	15.0
C9-7	11.8		12.0	24.3	19.7	164.7	15.0
C9-8	17.0		10.2	26.3	10.3	146.0	18.0
C9-9	4.3		6.1	19.5	5.0	86.3	1.5
C9-10	31.6		35.5	138.3	35.0	61.0	18.7
C9-11	10.2		0.7	11.3	6.0	27.7	2.3
C9-12	8.0		9.6	18.0	17.3	85.7	4.3
C9-13	12.2		24.6			114.0	10.3
C9-14	11.3	10.0	12.5	18.0	12.0	25.0	10.7
